# Increasing hexokinase 1 expression improves mitochondrial and glycolytic functional deficits seen in sporadic Alzheimer’s disease astrocytes

**DOI:** 10.1038/s41380-024-02746-8

**Published:** 2024-09-13

**Authors:** Simon M. Bell, Hollie Wareing, Francesco Capriglia, Rachel Hughes, Katy Barnes, Alexander Hamshaw, Liam Adair, Allan Shaw, Alicja Olejnik, Suman De, Elizabeth New, Pamela J. Shaw, Matteo De Marco, Annalena Venneri, Daniel J. Blackburn, Laura Ferraiuolo, Heather Mortiboys

**Affiliations:** 1 Sheffield Institute for Translational Neuroscience, School of Medicine and Population Health, University of Sheffield, 385a Glossop Rd, Sheffield, S10 2HQ UK; 2 NIHR Sheffield Biomedical Research Centre, University of Sheffield and Sheffield Teaching Hospitals NHS Foundation Trust, Sheffield, UK; 3 Neuroscience Institute, University of Sheffield, Firth Court, Sheffield, S10 2TN UK; 4 School of Chemistry, The University of Sydney, Sydney, NSW 2006 Australia; 5 Australian Research Council Centre of Excellence for Innovations in Peptide and Protein Science, The University of Sydney, Sydney, NSW 2006 Australia; 6 Department of Life Sciences, Brunel University London, Uxbridge, UK; 7 Department of Medicine and Surgery, University of Parma, Parma, Italy

**Keywords:** Neuroscience, Molecular biology, Biochemistry

## Abstract

Abnormalities in cellular metabolism are seen early in Alzheimer’s disease (AD). Astrocyte support for neuronal function has a high metabolic demand, and astrocyte glucose metabolism plays a key role in encoding memory. This indicates that astrocyte metabolic dysfunction might be an early event in the development of AD. In this paper we interrogate glycolytic and mitochondrial functional changes and mitochondrial structural alterations in patients’ astrocytes derived with a highly efficient direct conversion protocol. In astrocytes derived from patients with sporadic (sAD) and familial AD (fAD) we identified reductions in extracellular lactate, total cellular ATP and an increase in mitochondrial reactive oxygen species. sAD and fAD astrocytes displayed significant reductions in mitochondrial spare respiratory capacity, have altered mitochondrial membrane potential and a stressed mitochondrial network. A reduction in glycolytic reserve and glycolytic capacity is seen. Interestingly, glycolytic reserve, mitochondrial spare respiratory capacity and extracellular lactate levels correlated positively with neuropsychological tests of episodic memory affected early in AD. We identified a deficit in the glycolytic enzyme hexokinase 1 (HK1), and correcting this deficit improved the metabolic phenotype in sAD not fAD astrocytes. Importantly, the amount of HK1 at the mitochondria was shown to be reduced in sAD astrocytes, and not in fAD astrocytes. Overexpression of HK1 in sAD astrocytes increases mitochondrial HK1 levels. In fAD astrocytes HK1 levels were unaltered at the mitochondria after overexpression. This study highlights a clear metabolic deficit in AD patient-derived astrocytes and indicates how HK1, with its roles in both oxidative phosphorylation and glycolysis, contributes to this.

## Background

Alzheimer’s disease (AD) is the most common cause of dementia worldwide [1]. It is estimated that over 57.4 million people have the condition globally, with numbers expected to triple by 2050 [2]. Amyloid and tau aggregates within the brain are an important part of the pathology of AD. However, therapeutic agents designed to remove amyloid from the brain have shown mixed improvements in clinical outcomes [3–5]. Lecanemab, an amyloid clearing monoclonal antibody, reduces cognitive decline by 27% in patients with AD and was the first amyloid monoclonal antibody to reach all primary endpoints in clinical trials [6]. Donanemab, another monoclonal antibody, has recently been shown to have an even greater effect on cognitive decline [7]. As the approvals of antibody therapies are so recent, the full clinical potential is not clearly established. It is important to continue the investigation of other pathophysiological processes to understand the mechanisms contributing to neuronal injury in AD, and therefore provide potential adjunct therapies targeting different mechanisms to amyloid beta aggregation.

Metabolic changes are seen early within the brain of people with AD, and areas of high glucose metabolism are the same as those affected by amyloid aggregates, tau accumulation, and cortical atrophy [8–10]. This has led to the suggestion that metabolic failure, or reductions in metabolic efficiency of brain cells may be a key step in the development of AD [11, 12]. We and other groups have shown that the nervous system is not the only site of both mitochondrial dysfunction, and glycolytic change in AD [13]. Fibroblasts [14–18], platelets [19] and white blood cells [20] all show metabolic abnormalities. We have also shown that the parameters of mitochondrial function, i.e. mitochondrial spare respiratory capacity (MRSC) and mitochondrial membrane potential (MMP) in fibroblasts, correlate with the core neuropsychological changes seen early in AD [21]. Work using mouse models of AD has also shown that recovering mitochondrial function via inhibiting Cyclophilin D can prevent cognitive decline further highlighting the link between metabolic function and cognitive performance in AD [22].

Our previous work, and that of others, has shown that the capacity for both oxidative phosphorylation (OXPHOS) and glycolysis is impaired in the fibroblasts taken from patients with sporadic AD (sAD) and familial AD (fAD) [15, 18, 23–29]. The changes reported are associated with a reduction in MMP that may lead to adenosine triphosphate (ATP) reduction in the presence of physiological stress. If the metabolic capacity of the central nervous system becomes impaired, this could lead to the development of metabolic failure of the brain at times of increased energy expenditure. Interestingly, several established AD pathologies interact with the glycolysis pathway via the hexokinase enzymes. Hexokinase 1, the predominant isoform of the hexokinase enzyme within the brain is associated with the mitochondrial outer membrane. Dissociation of hexokinase from the mitochondrial membrane reduces its activity [30, 31], and affects ATP synthesis [32], with this effect exacerbated by amyloid [33] and glycogen synthase kinase-3 beta enzyme activity [34, 35]. Interleukin-1β a pro-inflammatory cytokine elevated in AD, reduces the expression of hexokinase, and causes dissociation from the mitochondrial membrane [36]. Therefore, metabolic processes, and specifically the function of the hexokinase 1 enzyme, interact with the key pathophysiological mechanisms of protein accumulation and neuroinflammation in AD. Characterising the metabolic changes occurring in AD will help to further our understanding of the pathology of this disease.

The vast majority of net brain metabolism occurs in neurons and astrocytes. These two cell types form a metabolic relationship, with the astrocyte playing a pivotal role in maintaining many cellular functions within the neuron [37–40]. Astrocytes provide neurons with metabolic substrates such as lactate, maintain the concentration of ion gradients and neurotransmitters at neuronal synapses, and can divert blood flow to areas of high metabolic activity, allowing increased oxygen and glucose uptake [41, 42]. Multiple studies have shown that pathological changes occur in astrocytes in AD [43–51]. Astrocyte glucose metabolism plays a key role in learning and memory, with astrocytic glycogen and astrocytic GABA release shown to be key substrates of memory encoding [52–56]. This dependent relationship between the neuron and the astrocyte puts specific metabolic demands on this cell type, meaning that any abnormalities in the function of astrocyte mitochondria, or the astrocyte’s ability to metabolise glucose will affect the function of both astrocytes and neurons.

Induced pluripotent stem cell (iPSC) technology has revolutionised neuroscience research, as it allows the generation of multiple nervous system cell linages from patients who have the disease under investigation [57–60]. Direct reprogramming of terminally differentiated cells can now be performed bypassing the embryonic stem cell phase [61, 62]. This reprogramming method allows cells to retain ageing markers from the parental somatic cells of origin [63], and this is an advantage when studying an age-related neurodegenerative disease such as AD.

In this study we have used induced neuronal progenitor cell (iNPC) technology [64–66] to derive astrocytes from patients with sAD and fAD (PSEN1 mutations). We uncovered significant changes in glycolysis and mitochondrial function, and identified alterations in these pathways which correlate with neuropsychological changes seen early in AD. Finally, we investigated the role that hexokinase 1 deficiency plays in the development of both mitochondrial and glycolysis deficits in AD, and whether correcting these deficits improves astrocyte metabolic output.

## Methods

### Patient demographics

Supplementary Tables 1 and 2 display demographic data for the sAD and fAD astrocyte lines and controls utilised in this study.

### Fibroblast reprogramming

**Fig. 1:**
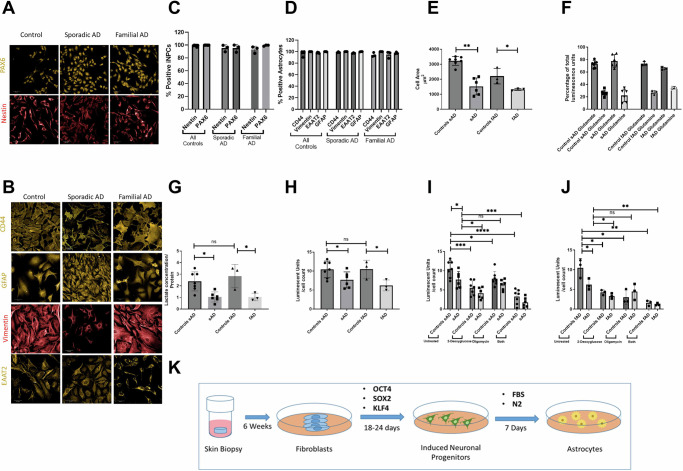
Astrocyte characterization. **A** Control, sporadic and familial AD astrocytes all express the neuronal progenitor cell markers PAX-6 and Nestin, scale bar 50 µm. **B** Control, sporadic and familial AD astrocytes display staining for CD44 (orange), GFAP (orange), Vimentin (red), and EAAT2 (orange), the procedure carried out at day 7 of differentiation, scale bar 50 µm. **C** shows the percentage of iNPC that stain for the markers PAX6 and Nestin. **D** Shows the percentage of astrocytes that stain for the markers CD44, GFAP, Vimentin and EAAT2. Panels **E**–**J** graph various parameters in astrocytes from sAD and relative controls, fAD and relative controls. Each experiment included 7 sAD controls, 6 sAD lines, 3 fAD control lines and 3 fAD lines. Data were analysed after at least 3 technical repeats and 3 biological repeats were performed in each experiment. In each technical repeat at least 300 cells were imaged and analysed per line. Each dot represents the mean of each line across 3 biological repeats. **E** Astrocyte cell area. *p < 0.05 **E** Intracellular fractions of glutamate and glutamine *p < 0.05. **F** Astrocyte extracellular lactate levels. *p < 0.05 and ***p < 0.001. **G** Total cellular ATP levels *p < 0.05 and **p < 0.01. **H**, **I** Total cellular ATP determination in the presence of 2-deoxyglucose and oligomycin *p < 0.05, **p < 0.01 and ****p < 0.0001. In all experiments AD astrocytes are compared with their relevant controls using t-tests. **K** The process of generating the patient-derived astrocytes. Skin biopsies are taken and cultured to generate fibroblasts over approximately 6 weeks. Fibroblasts are then transduced with retroviral vectors (OCT3, Sox2, and KLF4). Induced neuronal progenitor cells are generated over a period of 18–24 days, these cells are then cultured in a medium containing FBS and N2 that leads to the establishment of astrocyte cells after 7 days of differentiation.

Fibroblasts were set up from biopsy and cultured as previously described [15]. Fibroblasts were plated at a density of 250,000 cells per well. Twenty-four hours after plating, fibroblasts were transduced with retroviral vectors (OCT3, Sox2, and KLF4,) (ALSTEM). Forty-eight hours after transduction, cells were changed into iNPC media consisting of DMEM/F12, 1% N2, 1% B27 (Thermofisher Scientific), EGF (40 ng/mL) and FGF (20 ng/mL)(Peprotech). Once iNPC cultures were established, expression of the neuronal progenitor markers PAX-6 and Nestin (Fig. 1A) was confirmed using immunocytochemistry [62].

Astrocytes were differentiated from iNPCs using established protocols [63, 66]. iNPCs were seeded in a 10 cm dish coated with fibronectin (5 µg/mL, Millipore) in DMEM media (Lonza) containing 10% FBS (Biosera), and 0.3% N2 (Gibco) and differentiated for 7 days. After differentiation, expression of several astrocyte cell markers was confirmed using immunohistochemistry (GFAP, Vimentin, CD44, EAAT2, see Supplementary Table 3 for suppliers and concentrations and Fig. 1B for staining). All astrocyte lines were routinely tested for mycoplasma and confirmed negative.

### Immunocytochemistry

Details are provided in the supplementary material for immunohistochemistry methods and antibodies used.

### Glutamate uptake

An Abcam colorimetric Glutamate Assay kit (ab83389) was used to measure astrocyte glutamate uptake. The assay was performed as per manufacturer’s instructions using the standards and standard curve provided. Astrocytes were plated on a black 96-well plate (Greiner Bio-One) at a density of 10,000 cells/well at day 5 of differentiation. On day 7 of differentiation the astrocyte media was changed to Hank’s Balanced Salt Solution (HBSS) (Gibco), without calcium or magnesium, for 30 min. This medium was then changed to HBSS containing magnesium and calcium for 3 h with the medium also containing 100 µL of glutamate at a concentration of 1:1000. Samples were then collected as described by manufacturer and snap-frozen in liquid nitrogen. Glutamate measurement was performed as per kit protocol using a BMG Labtech plate reader.

### Total cellular ATP & ATP inhibitor assay

Cellular ATP levels were measured with the ATPlite kit (Perkin Elmer) as previously described [15]. Astrocytes were plated at a density of 5000 cells per well in a white Greiner 96-well plate (Greiner Bio-One) on day 5 of differentiation. ATP levels were corrected for cell number using CyQuant (ThermoFisher) kit [21]. The same assay was performed using the inhibitors of glycolysis (2-deoxyglucose 50 mM, Sigma) and OXPHOS (Oligomycin 1 µM, Sigma), or both (for 30 minutes) to assess the reliance on each metabolic pathway for total cellular ATP. Luminescence and fluorescence were read on a BMG Labtech plate reader.

### Mitochondrial membrane potential

Astrocytes were plated at a density of 2500 cells per well in a black Greiner 96-well plate (Greiner Bio-One) at day 5 after the start of differentiation. On day 7 of differentiation cells were incubated with tetramethlyrhodamine (TMRM) for 1 hour (concentration 80 nM) and Hoechst (concentration 10 nM, Sigma Aldrich) at 37˚C. Dyes were then removed, and astrocytes were maintained in Minimum Essential Medium whilst imaging using an INCELL Analyzer 2000 high-content imager (GE Healthcare). Twenty-five fields, with an average number of 500 cells per well at an emission/excitation spectrum of 543/604 m, were imaged. Mitochondrial morphological parameters and cell area were quantified using an INCELL developer protocol [67]. Parameters assessed included mitochondrial form factor (Form Factor = 1/(Perimeter^2^/4π.area), providing a measure of how round a mitochondria appears to be, and how interconnected the mitochondrial network is.

### Extracellular lactate

Extracellular lactate was measured using an L-Lactate assay kit (Abcam, ab65331). At day 7 of differentiation 1 µL of media was removed from a 10 cm dish containing confluent astrocytes and used in the assay as per the manufacturer’s instructions using standards provided.

## ATP substrate

The ATP substrate assay was used to investigate mitochondrial ATP production in the presence of complex I and II substrates. 500,000 cells were collected at day 7 of astrocyte differentiation. Methods have been previously described by Manfredi et al. [68]. In brief, cells were suspended in 250 µL of buffer A (150 mM KCl, 25 mM Tris HCl, 2 mM EDTA, 0.1% BSA, 10 mM K_3_PO_4_, and 0.1 mM MgCl, pH 7.4). Cells were then permeabilised with histone 2 ug/mL for 5 min. After permeabilization, 5 volumes of buffer A were added to the cell suspension. The suspension was then centrifuged for 5 min at 17,000 × *g*. Cells were then resuspended in 150 µL of buffer A. 550 µL of buffer A were added to the remaining 100 µL of suspension for use in the substrate assay.

A PHERAstar plate reader (BMG Labtech) in luminescence mode was used. A background luminescence reading was made of each well on the assay plate containing 160 µL cell suspension then one of either the complex I substrates (malate 1.25 mM and galactose 1.25 mM), or complex II substrates (succinate 1.25 mM, rotenone 1 µM complex I inhibitor) were added. After baseline kinetics were measured, the machine was paused and adenosine diphosphate (ADP, 4 µM) and 10 µL of the ATP substrate solution (containing luciferin/luciferase), described above in the ATP assay section, were added to each well. The kinetics assay was then resumed, and measurements of substrate use were made for the next 30 min. The gradient of the kinetic curve was calculated and normalised to protein content using a Bradford assay (details of which are in supplementary material).

### Metabolic flux

#### Mitochondrial stress test

Astrocyte OXPHOS was assessed by measuring oxygen consumption rates (OCR) using a 24-well Agilent Seahorse XF analyzer. Astrocytes were plated at a density of 10,000 cells per well at day 5 of differentiation. At day 7 of differentiation, astrocytes were switched to XF media (Agilent) and then assessed using the previously described *Mitochondrial Stress Test Protocol* [15].

#### Glycolysis stress test

Astrocyte glycolysis was measured using the glycolysis stress test protocol on a 24-well Agilent Seahorse XF analyzer. Astrocytes were plated at a density of 10,000 cell per well at day 5 of differentiation. At day 7 of differentiation, as with the mitochondrial stress test, astrocytes were transferred to XFmedia (Agilent), and glycolysis was assessed. The glycolysis stress test was used to assess glycolysis, as described previously [21]. Measurements were normalised to cell count, measured using Hoechst nuclear staining, imaged on an InCell Analyser high content imager.

### Glucose uptake

Glucose uptake by astrocytes was measured using the Glucose Uptake Assay Kit (Fluorometric) (Abcam, ab136956). Astrocytes were plated at a density of 2500 cells per well in a 96-well black (Greiner Bio-One) plate on day 5 of differentiation. The assay was performed on day 7 of differentiation as per protocol. Measurements of fluorescence at λ_Ex/Em_ = 535/587 nm were then taken using PHERAstar plate reader (BMG Labtech).

### Glutamine/glutamate assessment

A Glutamine/Glutamate-Glo™ Assay (Promega) was used to measure intracellular concentrations of glutamine and glutamate. A 10 cm dish of astrocytes (≈2,000,000 cells) was immersed in inactivation solution (2mLs of HCl 0.3 N and 1 mL PBS) for 5 minutes on day 7 of differentiation. After this the dish was scraped and 1 mL of Tris solution (2-amino-2-(hydroxymethyl)-1,3-propanediol, 450 mM at pH 8.0) was added to the cells. 200 µL of this solution was then added to 200 µLs of PBS. 25 µL of this dilution were then placed in a well of a white 96-well plate (Greiner Bio-One). After sample preparation, the assay was performed as per the protocol provided by Promega with measurements taken using PHERAstar plate reader (BMG Labtech) using standards provided.

### Mitochondrial complexes

Mitochondrial complex assays were performed for mitochondrial complexes I (ab109721), II (ab109908) and IV (ab109909). Assays were carried out as per the manufacturer’s instructions (Abcam). Approximately 2,000,000 astrocytes per experiment were used for each complex assay. Once complex assay values had been assessed, values were normalised to sample protein content using a Bradford assay (details in Supplementary materials).

### Hexokinase activity

Hexokinase activity was measured using the Abcam (ab136957) Hexokinase Assay Kit (Colorimetric) as per the manufacturers’ protocol. Astrocytes were harvested on day 7 of differentiation. In brief, approximately 2,000,000 astrocytes were collected and homogenised using the assay buffer. Samples were centrifuged at 12,000 rpm, for 5 minutes and then kept on ice until assay assessment. Once activity values had been assessed, values were normalised to sample protein content determined via a Bradford assay, details in supplementary materials.

### Quantitative Polymerase Chain Reaction (qPCR)

Supplementary Table 4 describes the qPCR techniques and gene primer sequences used.

### Mitochondrial isolation

Mitochondria were isolated using differential centrifugation. Two confluent dishes were washed twice in cold PBS, harvested by scraping, and pelleted at 600 × *g* for 10 min at 4 °C. Cells were lysed by adding 1 ml of cold mitochondria isolation buffer (MIB: 250 mM sucrose, 3 mM EDTA, 20 mM HEPES, pH 7.5) and homogenised by 10 strokes using a glass to glass handheld homogenised followed by 10 strokes using a Thomas homogenizer with a motor driven Telfon pestle. Homogenate was centrifuged at 600 × *g* for 10 min at 4 °C and the supernatant was subsequently spun at 15,000 × *g* for 10 min at 4 °C. The low-speed pellet was re-homogenised and the supernatant spun at 15,000 × *g* for 10 min at 4 °C. The mitochondrial pellets were suspended in a small volume of MIB and protein concentration was determined by the Bradford assay (details of which are in the supplementary material). The mitochondria were stored at −80 °C.

### Mitochondrial reactive oxygen species

Mitochondrial reactive oxygen species (ROS) were quantified using the mitochondrially-targeted fluorescent redox sensor NpFR2 [69]. Astrocyte cells were plated at a density of 2500 cells per well on a 96 well plate and incubated with NpFR2 at a concentration of 20 µM for 30 min prior to imaging. NpFR2 fluorescence intensity was visualised using an Opera Phenix high content imager (λ_Ex/Em_ 488 nm/530 nm) (Perkin Elmer). Imaging protocols were as described previously [70].

### Western blots

Supplementary Table 3 describes the western blot techniques and antibodies used.

### Viral transduction

Day 4 of astrocyte differentiation, astrocytes were transduced with a hexokinase adenoviral vector (Ad-h-HK1). The adenovirus (AdV) was purchased from VectorBiolabs (RefSeq#: BC008730). All hexokinase AdV experiments were performed with a multiplicity of infection (MOI) of 40. Astrocytes were transduced with the virus for 72 h and then measurements of mitochondrial and glycolytic function were made. An AdV containing a scramble gene (VectorBiolabs) was also plated for each experiment with a MOI of 40 also used.

### Statistical analysis

For metabolic datasets comparisons between AD groups and the control groups were carried out using t-tests. Normality testing was undertaken in GraphPad Prism prior to running statistical tests. A Pearson correlation was performed for metabolic neuropsychological correlations. Statistics were calculated through GraphPad v8 software and IBM SPSS statistics version 29.

## Results

### Astrocytes derived from sAD/fAD patients display reduced total cellular ATP and extracellular lactate

iNPCs from control, sAD and fAD patients all expressed the neuronal precursor markers Nestin and PAX6 in greater than 95% of all cells (Fig. 1A, C), consistent with previous data demonstrating that direct conversion results in a homogeneous iNPC population [63]. Upon differentiation, all astrocyte lines expressed CD44, GFAP, Vimentin, and EAAT2 in greater than 90% of cells (Fig. 1B, D), confirming successful differentiation of iNPCs into astrocytes, as previously published [66, 71, 72]. Supplementary Fig. 1 highlights additional astrocyte characterisation performed confirming the retention of some aging markers from parental fibroblasts to reprogrammed astrocytes, as described previously [63]. Both sAD (difference in mean area 1015 µm^2^ SEM ± 355.5, *p* = 0.003) and fAD astrocytes (difference in mean area 1319 µm^2^ SEM ± 887.0, *p* = 0.0424) had a statistically significant smaller cell area compared to control astrocytes (sporadic controls mean area 3221 µm^2^, familial controls mean area 2206 µm^2^) (Fig. 1E).

Functionally, all astrocyte lines had similar intracellular proportions of glutamine and glutamate (Fig. 1F). Extracellular lactate levels were reduced in both sAD (56% reduction SEM ± 15%, *p* = 0.0043) and fAD astrocytes (63% reduction SEM ± 21%, *p* = 0.0421) when compared with their control groups (Fig. 1G).

Considering the important role of astrocytes in brain energy metabolism, we decided to measure the capacity of fAD and sAD astrocytes to produce ATP compared with astrocytes derived from healthy individuals. A significant reduction in total cellular ATP was seen in sAD (32% reduction SEM ± 11.2%, *p* = 0.0158) and fAD (41.0% reduction SEM ± 15.2%, *p* = 0.050) astrocytes (Fig. 1H). Consistent with the notion that astrocytes rely on glycolysis more than OXPHOS for energy production [40], a greater proportion of total cellular ATP was produced via glycolysis when compared with OXPHOS in all astrocyte lines (Fig. 1I, J). Astrocyte total cellular ATP had a mean reduction of 45% across all three astrocyte groups when glycolysis was inhibited (control 47%, sAD 43% and fAD 46% reduction), whereas a mean reduction of 20% in total cellular ATP was seen when oligomycin was added to inhibit OXPHOS (controls 24%, sAD11.8% and fAD 28% reduction, Fig. 1I, J). Figure 1K shows the reprogramming procedure.

### AD astrocytes have altered mitochondrial morphology and MMP. Oxidative phosphorylation is altered in sAD/fAD astrocytes, with a specific reduction in mitochondrial spare respiratory capacity

**Fig. 2:**
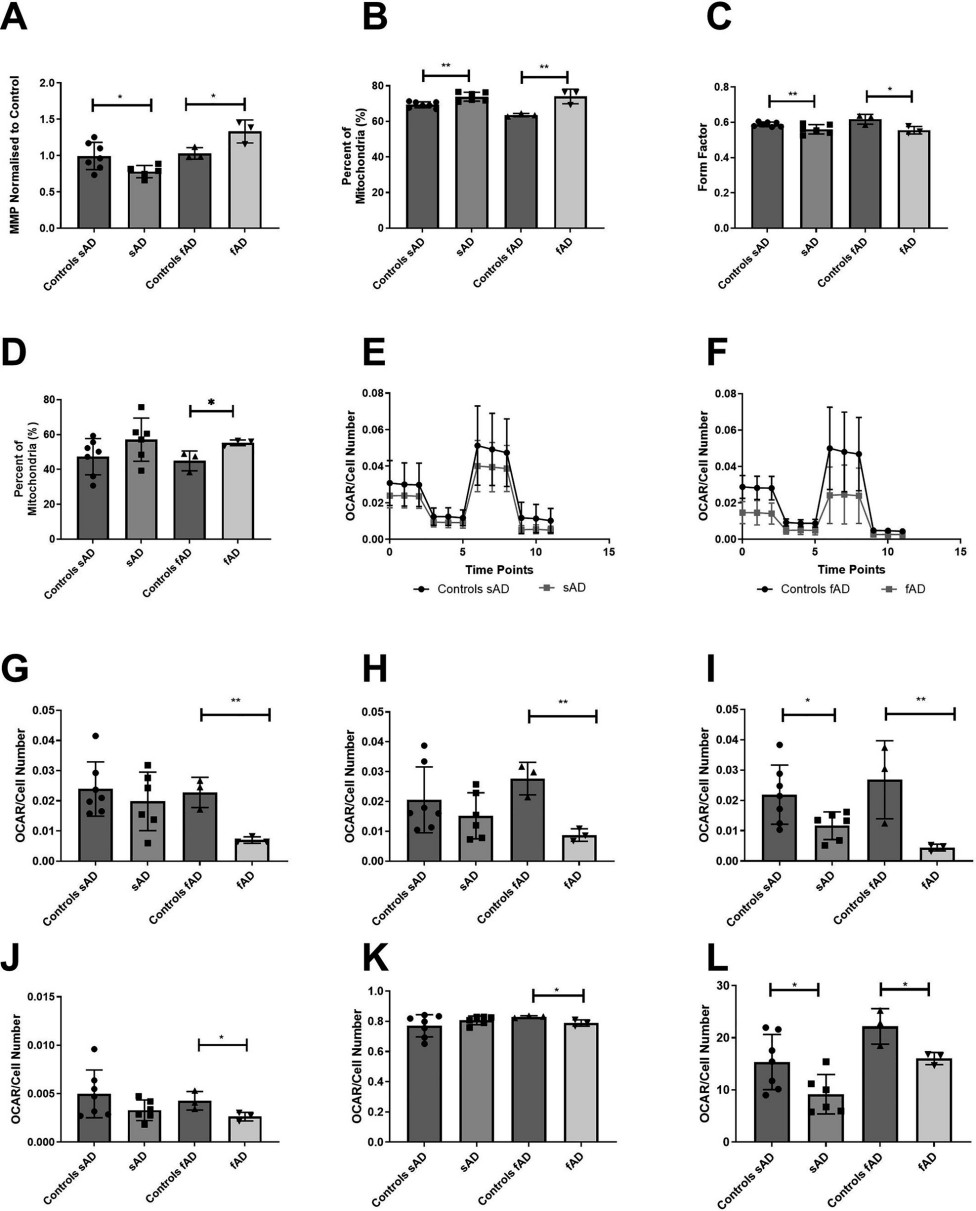
Astrocyte mitochondrial morphology and oxidative function. **A** Astrocyte mitochondrial membrane potential was shown to be significantly reduced in sAD astrocytes but significantly increased in fAD astrocytes. *p < 0.05. **B** Percentage of long mitochondria, as a proportion of the whole mitochondrial population, was significantly increased in both fAD and sAD compared with controls. **p < 0.01. **C** Form factor was significantly reduced in both fAD and sAD astrocyte types.*p < 0.05 and **p < 0.01. **D** Perinuclear mitochondria percentage as a proportion of the whole mitochondrial network. *p < 0.05. **E** Sporadic AD astrocyte OCR trace. **F** Familial AD astrocyte OCR trace. **G** ATP Linked Respiration. **p < 0.01. **H** Basal Mitochondrial Respiration Basal. **p < 0.01 **I** Mitochondrial Spare Respiratory Capacity. *p < 0.05 and **p < 0.01 **J** Proton leak. *p < 0.05. **K** Coupling Efficiency. *p < 0.05. **L** OCR/ECAR Ratio *p < 0.05, **p < 0.01. In all experiments AD astrocytes are compared with their relevant controls using t-tests. Each experiment included 7 sAD controls, 6 sAD lines, 3 fAD control lines and 3 fAD lines. Data were analysed after at least 3 technical repeats and 3 biological repeats were performed in each experiment.

To investigate further the observed reduction in ATP levels, mitochondrial morphology and functionality were assessed. sAD astrocytes showed a significant reduction in the MMP when compared with controls (22% reduction SEM ± 9.9%, *p* = 0.05), whereas fAD astrocytes had a higher MMP (30% increase, *p* = 0.041 SEM ± 10%) (Fig. 2A). sAD and fAD astrocytes had an increased number of elongated mitochondria (sAD Astrocytes 6.4% increase, *p* = 0.002, SEM ± 1.1%, fAD astrocytes 16% increase, *p* = 0.0127 SEM ± 2.4%) (Fig. 2B), and a significantly reduced form factor which, due to the equation used to calculate this (see Methods), indicates that the mitochondrial network is more interconnected. (sAD astrocytes 4.7% reduction SEM ± 1%, *p* = 0.029, fAD astrocytes 10% SEM ± 2% reduction, *p* = 0.036; Fig. 2C).

A trend towards an increase in the percentage of perinuclear mitochondria was seen in sAD astrocytes (*p* = 0.150), with fAD astrocytes showing a significant increase in perinuclear mitochondria (23% increase SEM ± 10.3%, *p* = 0.039, SEM ± 3.4%; Fig. 2D). The percentage of small mitochondria in the mitochondrial network, and mitochondrial number were analysed, but no differences were seen in these parameters (data not shown).

Figure 2E, F shows OCR for sporadic and familial AD astrocytes respectively. These panels show mitochondrial oxygen consumption after the addition of electron transport chain (ETC) inhibitors allowing assessment of the different aspects of OXPHOS. ATP linked respiration (69% reduction SEM ± 13%, *p* = 0.005) and basal mitochondrial respiration (68.4% reduction SEM ± 12%, *p* = 0.004) were both significantly lower in fAD astrocytes (Fig. 2G, H). In sAD astrocytes, although a trend was seen for reduced basal mitochondrial respiration and ATP-linked respiration, these changes were not significant (Fig. 2G, H). In addition, MSRC was shown to be significantly reduced in both sAD (46.6% reduction SEM ± 19.8%, *p* = 0.038) and fAD astrocytes (83.5% reduction SEM ± 27.8%, *p* = 0.040) (Fig. 2I). fAD astrocytes showed a significant reduction in proton leak, (38.5% reduction SEM ± 21.8%, *p* = 0.050), whereas sAD astrocytes only showed a trend towards a reduction (Fig. 2J). Consistently, no significant difference was seen in coupling efficiency or respiratory control ratio in sAD astrocytes, but a significant reduction in coupling efficiency was seen in fAD astrocytes (5% reduction SEM ± 1.4%, *p* = 0.032, see Fig. 2K). In both sporadic and familial astrocytes, a reduction in the OCR/ECAR ratio, which indicates the metabolic status of the cells, was seen, possibly suggesting a metabolic shift in AD astrocytes (sAD reduction 40% SEM ± 16.8%, *p* = 0.0368, fAD 27.5% reduction SEM ± 9.3%, *p* = 0.041) (Fig. 2L).

### sAD/fAD astrocyte mitochondria display increased mitochondrial ROS production, reduced complex I linked ATP production and increased complex I activity

**Fig. 3:**
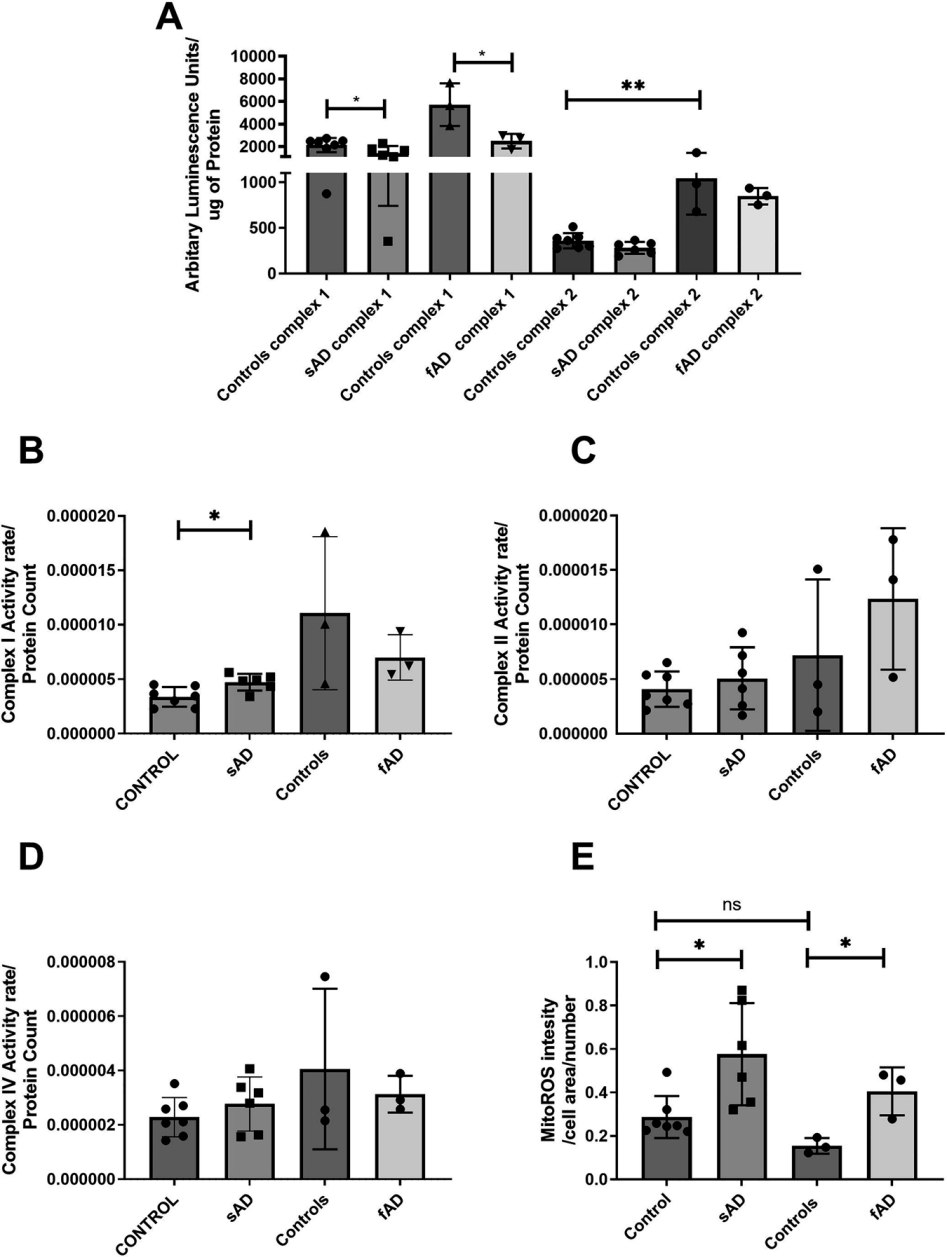
Mitochondrial electron transport chain complex assessment*.* **A** Assessment of mitochondrial ATP production in an abundance of complex I & II substrates. *p < 0.05, ***p < 0.001 and ****p < 0.0001. **B** Direct assessment of complex I activity. *p < 0.05. **C** Direct assessment of complex II activity. **D** Direct assessment of complex IV activity. **E** Astrocyte mitochondrial reactive oxygen species production assessment. *p < 0.05 In all experiments AD astrocytes are compared with matched controls using t-tests. Each experiment included 7 sAD controls, 5 sAD lines, 3 fAD control lines and 3 fAD lines. Data were analysed after at least 3 technical repeats and after 3 biological repeats were performed in each experiment.

As both sAD and fAD astrocytes have a deficit in respiration suggesting deficiencies in OXPHOS function, we measured the ATP production linked to specific ETC complexes. Both sAD and fAD astrocytes showed a significant reduction in mitochondrial ATP production when supplied with complex I substrates (sAD: 34% reduction SEM ± 15.3%, *p* = 0.046, fAD: 56% reduction SEM ± 20.1%, *p* = 0.0485) (Fig. 3A). No differences in mitochondrial ATP production were seen when complex II substrates were supplied to the permeabilised cells, suggesting no deficit in complex II activity or downstream complexes in the respiratory chain (Fig. 3A). When maximal complex I activity was measured in isolation, activity was significantly higher in sAD astrocytes (43.4% increase, SEM ± 13.1% *p* = 0.038, Fig. 3B), with no difference seen in fAD complex I activity when compared with controls (Fig. 3B). Complex II activity showed no difference in sAD astrocytes with a trend to an increase seen in fAD complex II activity (Fig. 3C). No change in complex IV activity was seen in AD astrocytes (Fig. 3D). Finally, to assess the efficiency of the mitochondrial ETC we measured mitochondrial reactive oxygen species (ROS) levels. A significant increase in ROS production was seen in sAD (200% increase, SEM ± 31.1%, *p* = 0.012) and fAD (260% increase, SEM ± 26.6%, *p* = 0.020) astrocytes (Fig. 3E).

### Astrocyte glycolytic function is impaired in AD astrocytes

**Fig. 4:**
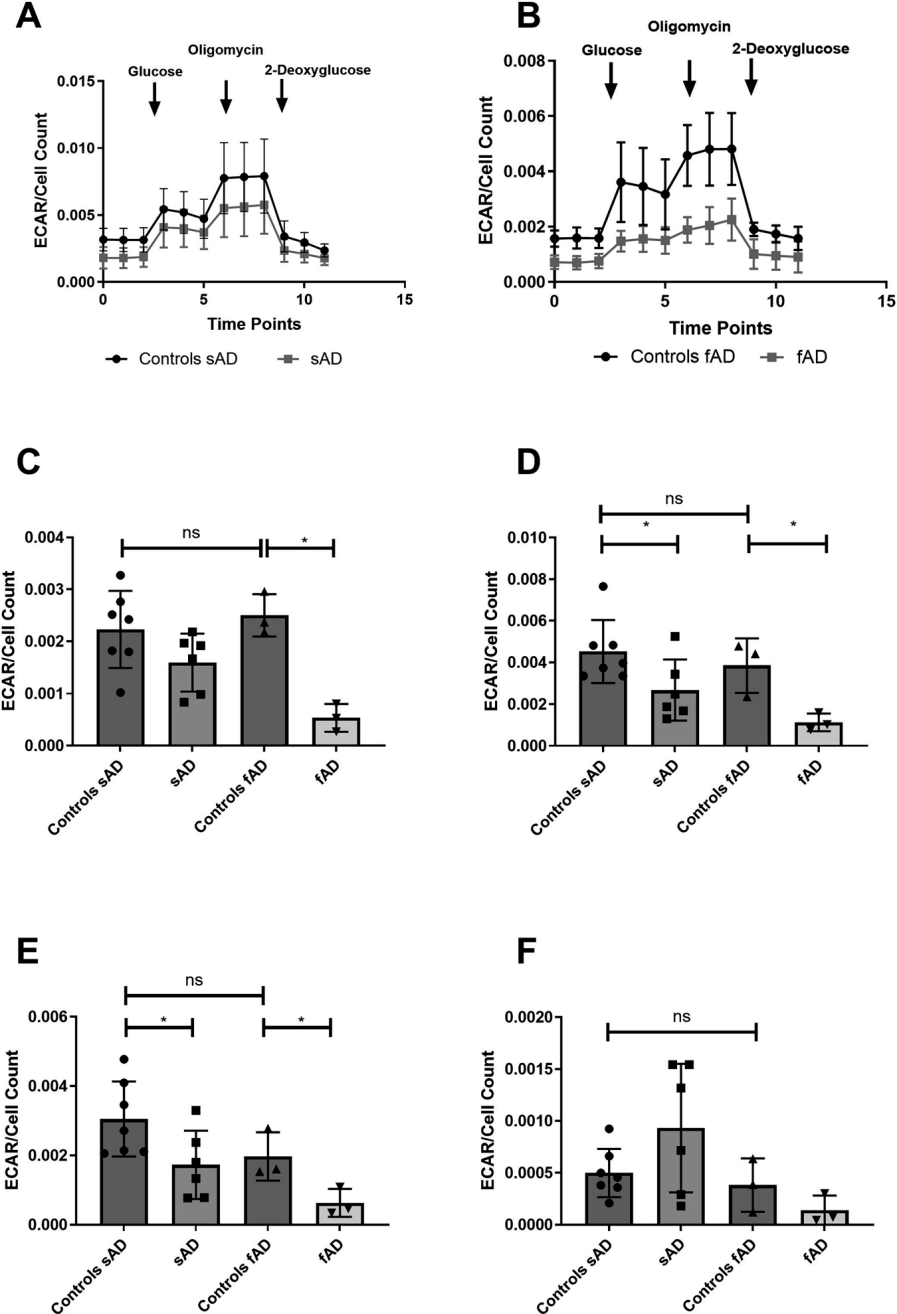
Astrocyte glycolysis assessment*.* **A** ECAR trace sporadic astrocytes **B** ECAR trace familial Astrocytes. **C** Glycolytic rate *p < 0.05. **D** Glycolytic Capacity. *p < 0.05. **E** Glycolytic Reserve. *p < 0.05. **F** Non-glycolytic acidosis in all experiments AD astrocytes are compared with matched controls using t-tests. Each experiment included 7 sAD controls, 5 sAD lines, 3 fAD control lines and 3 fAD lines. Data were analysed after at least 3 technical repeats and after 3 biological repeats were performed in each experiment.

Total ATP measurements indicated that glycolysis inhibition led to the most profound decrease in ATP production in both fAD and sAD astrocytes. To further investigate glycolytic function, we next measured glycolysis using the glycolysis stress test (Fig. 4A, B show the glycolysis stress test trace performed in sAD and fAD astrocyte lines and comparative controls). A significant reduction in glycolysis rate was seen in fAD astrocytes (78.6% reduction, *p* = 0.0022, SEM ± 11%), with more variation between sAD patient lines seen (28.2% reduction, *p* = 0.1124) (Fig. 4C). A significant reduction in glycolytic capacity was measured in both sAD (40.9% reduction, *p* = 0.0466, SEM ± 18.1%) and fAD astrocytes (70.6% reduction, *p* = 0.0263, SEM ± 20.5%) (Fig. 4D). In addition, glycolytic reserve was significantly reduced in sAD (43% decrease, *p* = 0.043, SEM ± 16.4%) and fAD (68% decrease, *p* = 0.044, SEM ± 24.2%) astrocytes (Fig. 4E), while no significant difference in non-glycolytic acidosis was seen in AD astrocytes (Fig. 4F).

### sAD/fAD astrocytes have impaired glycolytic function at multiple stages in the glycolytic pathway

**Fig. 5:**
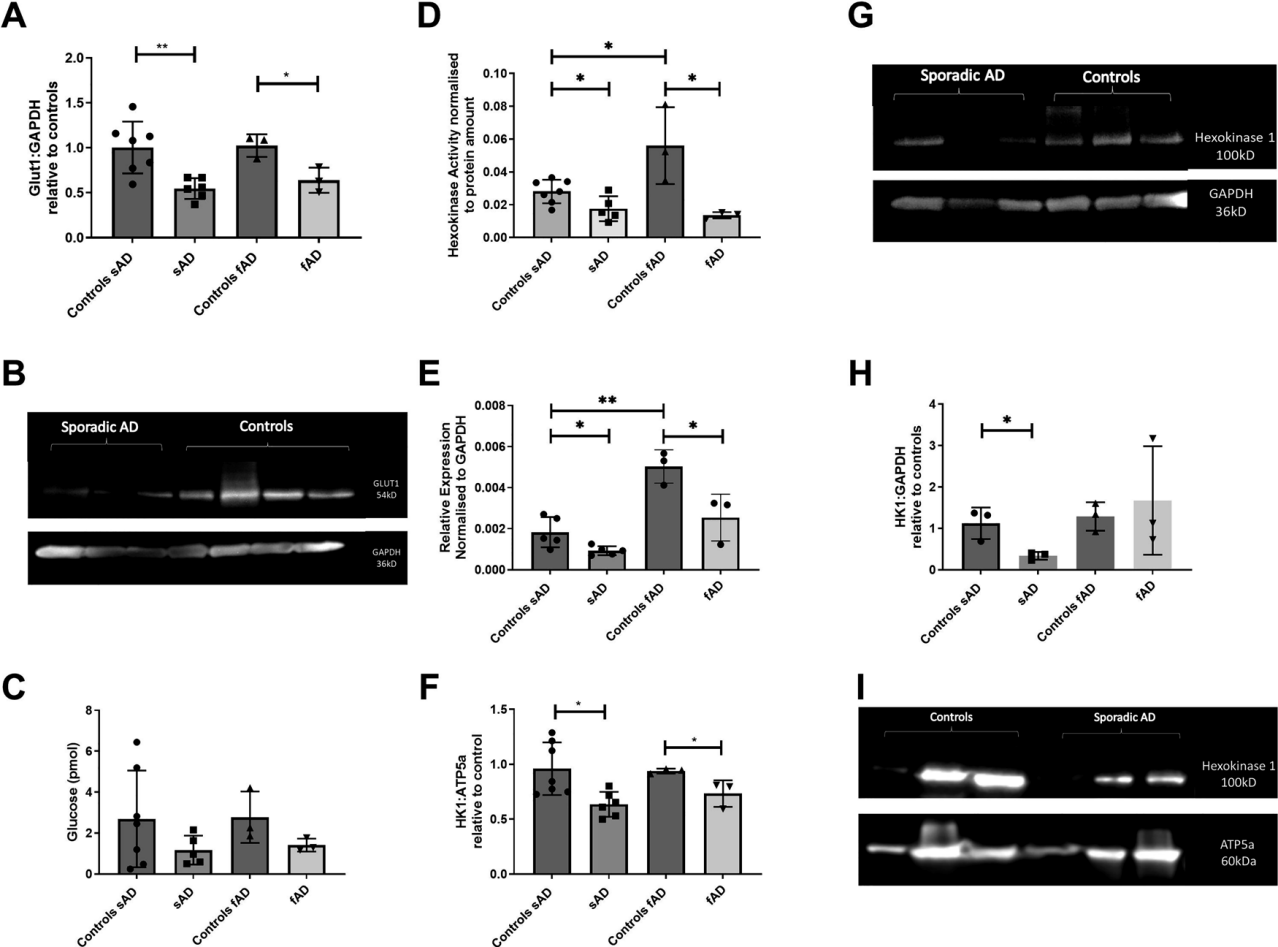
Astrocyte glucose uptake, hexokinase expression and activity. **A** GLUT1 astrocyte protein expression *p < 0.05, **p < 0.01. **B** Representative western of GLUT1 blot **C** Astrocyte 2-deoxyglucose uptake. **D** Hexokinase 1 activity *p < 0.05. **E** Hexokinase mRNA expression *p < 0.05, **p < 0.01. **F** Hexokinase protein expression *p < 0.05. **G** Representative blot of hexokinase protein expression. **H** Relative expression of Hexokinase 1 to ATP5a mitochondrial protein in enriched mitochondrial fraction. **I** representative blot of relative hexokinase 1 expression. In all experiments AD astrocytes were compared with matched controls using t-tests. Each experiment included 7 sAD controls, 5 sAD lines, 3 fAD control lines and 3 fAD lines, except for figures H&I were the three sAD with the lowest hexokinase 1 expression were used. Data were analysed after at least 3 technical repeats and 3 biological repeats were performed in each experiment.

The striking impairment of glycolysis in the AD astrocytes led us to investigate a major mechanism upstream of glycolysis, glucose uptake rates. This parameter was measured using 2-deoxyglucose and quantifying the amount of astrocyte glucose transporters. Glucose transporter protein expression (GLUT1, GLUT2 & GLUT4) was measured via western blot. GLUT1 receptor protein expression was shown to be reduced in sAD (50% reduction, SEM ± 18%, *p* = 0.016) and fAD astrocytes (79% reduction, SEM ± 11%, *p* = 0.003) (see Fig. 5A, B shows a representative blot). We could not identify expression of GLUT2 or 4 receptors in any of the astrocytes using western blot analysis. The observed reduction in GLUT1 led to the hypothesis that sAD and fAD astrocytes would display impaired glucose uptake. Although a trend to reduced 2-deoxyglucose uptake was seen, significant interline variability in both control astrocytes and AD astrocytes was observed in glucose uptake, and hence no group changes were found (Fig. 5C). To investigate further the glycolytic dysfunction, we sought to assess both the levels and activity of hexokinase 1 that represents a pivotal and rate limiting enzyme. Hexokinase 1 activity was significantly reduced in sAD (37% reduction, SEM ± 15.3%, *p* = 0.034) and fAD (75% reduction, SEM ± 24%, *p* = 0.035) astrocytes (Fig. 5D). Hexokinase mRNA expression was assessed using qPCR that also showed a reduction in both sAD (49% reduction, SEM ± 18.5%, *p* = 0.029) and fAD (49.8% reduction, SEM ± 16%, *p* = 0.036) astrocytes (Fig. 5E). Hexokinase protein expression measured using western blot analysis revealed reduced expression in sAD (48.6% reduction SEM ± 21.4%, *p* = 0.045) and fAD (38.6% reduction, SEM ± 12.8%, *p* = 0.041) astrocytes (see Fig. 5F, G for a representative blot). Finally, we assessed Hexokinase 1 protein localisation within the astrocytes (Fig. 5H, I for representative blot) and identified that in sAD astrocytes there was a significant reduction in Hexokinase 1 in the mitochondrially enriched fraction (70% reduction, SEM ± 20%, *p* = 0.025), but no reduction in fAD when compared to controls (Fig. 5H). The purity of the mitochondrial enriched fractions is shown using ATP5a in Supplementary Fig. 2. We did not identify a significant difference in GAPDH, ATP5a expression, or mitochondrial number in any groups (Supplementary Fig. 2). Conversely there was a trend to a larger proportion of hexokinase 1 in the cytoplasm in sAD, when compared to controls (Supplementary Fig. 2). This relationship was not seen when fAD astrocytes were compared to controls.

### Astrocyte metabolic output and capacity biomarkers correlate with neuropsychological indices in sporadic AD astrocytes but not with hexokinase 1 activity

In a previous report we investigated if MRSC and MMP measured in sAD fibroblasts correlated with neuropsychological markers of AD [21]. Since glycolytic function is more significantly impaired in sAD derived astrocytes than in fibroblasts [21], we investigated whether glycolytic reserve and extracellular lactate correlated with neuropsychological abnormalities present in sAD. Neuropsychological data were only available for sAD astrocytes and their controls. Semantic fluency (a measure of semantic memory), immediate and delayed episodic recall were assessed as these neuropsychological markers were previously investigated and are affected in the early stages of AD [73–76]. Correlations are shown in Supplementary Figs. 3 and 4. As described previously in our report using fibroblasts from the same participants, we assessed the correlations after controlling for brain reserve, years of education and participant age. Only correlations that were statistically significant were assessed. After controlling for the 3 factors, positive correlations remained between immediate episodic recall and glycolytic reserve (correlation = 0.766, *p* = 0.016), delayed episodic recall and glycolytic reserve (correlation = 0.700, *p* = 0.036), immediate episodic recall and extracellular lactate levels (correlation = 0.749, *p* = 0.020) and delayed episodic recall and extracellular lactate (correlation = 0.677, *p* = 0.045). Supplementary Table 5 shows these correlations. Conversely hexokinase 1 activity was not found to correlate significantly with any of the neuropsychological test results (Supplementary Fig. 5).

### Restoring hexokinase expression improves markers of astrocyte metabolic output and reduces mitochondrial ROS in sAD astrocytes

**Fig. 6:**
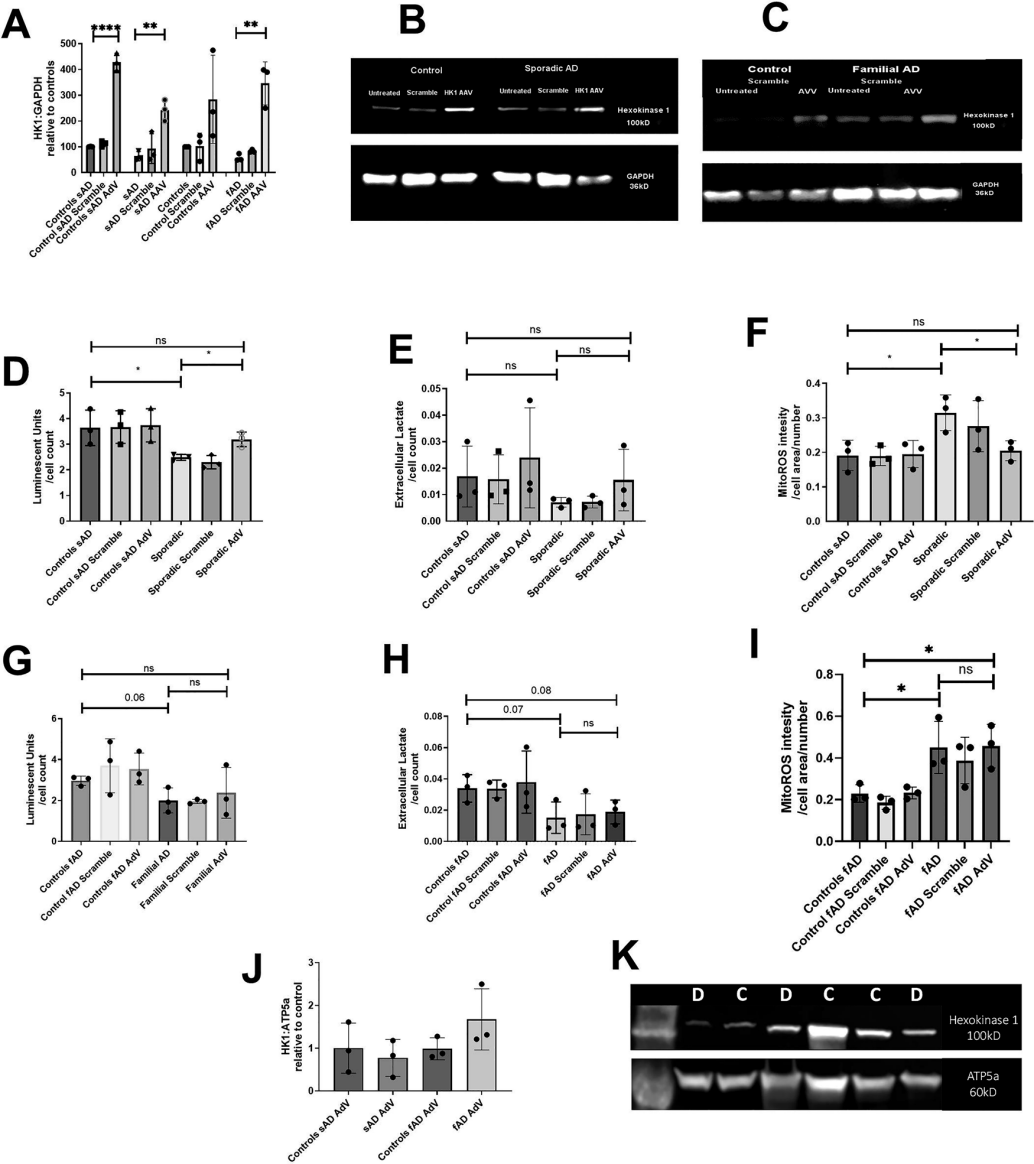
Transduction with a hexokinase-containing AdV vector restores metabolic outputs in sAD astrocytes but not fAD. **A** Hexokinase protein expression after transduction with the hexokinase containing AdV. *p < 0.01, **B** Representative western for hexokinase expression in sAD astrocytes and controls. **C** Representative western for hexokinase expression in fAD astrocytes and controls. **D** Total cellular ATP levels after AdV transduction in sAD. *p < 0.05 **E** Extracellular lactate levels after AdV transduction in sAD lines. **F** Astrocyte mitochondrial ROS production after AdV transduction sAD lines. *p < 0.055, and **p < 0.01. **G** Total cellular ATP levels after AdV transduction in fAD. **H** Extracellular lactate levels after AdV transduction in sAD lines. **I** Astrocyte mitochondrial ROS production after AdV transduction fAD lines. *p < 0.05. **J** Relative expression of hexokinase in enriched mitochondrial fractions after transduction with AdV **K** representative blot in this blot “C” refers to controls and “D” refers to sAD lines. In all experiments AD astrocytes are compared with matched controls using t-tests. Each experiment included 3 sAD controls, 3 sAD lines, 3 fAD control lines and 3 fAD lines. Data were analysed after at least 3 technical repeats and after 3 biological repeats were performed in each experiment.

As hexokinase 1 function represents a link between glycolysis and mitochondrial energetic function, we assessed whether correcting the deficit in astrocyte hexokinase expression improved astrocyte metabolic outputs. We selected the 3 sAD astrocyte lines with the largest hexokinase deficit to transduce with an AdV vector expressing hexokinase 1. Expression of the hexokinase protein increased by 160% in sAD astrocytes, *p* = 0.024, SEM ± 42%, when compared to the scramble condition and by 315% in fAD astrocyte lines, *p* = 0.0276, SEM ± 57% when compared to the scramble condition (Fig. 6A–C). Hexokinase 1 levels were significantly increased after overexpression in the sAD control astrocytes by 283%, SEM 40%, p = 0.0024, however hexokinase 1 expression increased by 184% in fAD controls SEM ± 102%% which was not significant due to inter line variability (Fig. 6A, see Fig. 6B, C for representative blots). In sAD astrocyte lines transduction with the hexokinase 1 containing AdV led to a significant increase in total cellular ATP (28.1%% increase, SEM ± 7.1%, *p* = 0.017, Fig. 6D), a non-significant increase in extracellular lactate (118.3% increase SEM ± 95%, *p* = 0.283, Fig. 6E) and a significant reduction in mitochondrial ROS (34.9% decrease SEM ± 10.7%, *p* = 0.032, Fig. 6F). Interestingly, in fAD astrocytes, transduction with the hexokinase 1 containing AdV did not increase total cellular ATP (Fig. 6G), or extracellular lactate levels (Fig. 6H) or lead to a significant reduction in mitochondrial ROS (Fig. 6I). We assessed if hexokinase 1 in the mitochondrially enriched fraction was altered by the overexpression of hexokinase 1. We found in sAD astrocytes, the hexokinase 1 levels were restored in the mitochondrially enriched fraction, whereas in fAD astrocytes, no change in the amount of hexokinase 1 was seen (Fig. 6J, representative blot 6 K). Mitochondrial morphological parameters that were, abnormal in sAD and fAD astrocytes were not improved with AdV transduction (Supplementary Figure 6). Finally, we assessed if the hexokinase 1 expression correlated with either the baseline ATP or mitochondrial ROS levels or indeed the change in ATP or mitochondrial ROS when hexokinase 1 is overexpressed. The lines with the lowest HK1 expression also have the lowest ATP levels and the highest mitochondrial ROS levels. Indeed, the lines with the lowest basal HK1 expression also have the largest change in ATP and mitochondrial ROS when hexokinase 1 is overexpressed (Supplementary Fig. 7).

## Discussion

In this study we have shown that astrocytes derived from patients with both sAD and fAD have deficits in glycolytic function and in mitochondrial structure and function. These abnormalities are strikingly similar between sAD and fAD astrocytes. Some of these abnormalities correlate with neuropsychological abnormalities seen early in AD. Hexokinase 1 is a key enzyme in the glycolytic pathway that links the major metabolism pathways of glycolysis and OXPHOS. We find that overexpression of hexokinase 1 remarkably restores most metabolic abnormalities in the sAD astrocytes, suggesting that in sAD, hexokinase 1 activity might be a therapeutic target to restore astrocyte glucose metabolism, which may have an effect on cognitive performance. However, hexokinase 1 overexpression does not restore fAD metabolic function, suggesting divergent pathways leading to these metabolic abnormalities in these two patient groups. The hexokinase 1 expression at the mitochondria appears to be key in this rescue in sAD and lack of rescue in fAD astrocytes. Presenilin 1 has been shown to regulate mitochondrial membrane composition and contacts with the endoplasmic reticulum [46, 77]; our data suggest differing mechanisms leading to mitochondrial and glycolytic abnormalities seen in sAD and fAD cells. Presenilin 1 mutations likely have a direct effect at the mitochondrial membrane, meaning the phenotypes cannot be altered by hexokinase 1 expression but rather a targeted approach to PSEN1 may have to be employed. This, however, is the beyond the scope of present work.

Our work builds upon limited studies available to date investigating metabolism in astrocytes derived from AD patients. One of the few published studies found a decreased rate of glycolysis, glycolytic reserve and lower extracellular lactate levels in astrocytes derived from 3 PSEN1 mutation carriers when compared with controls [78]. In a further study using iPSC derived astrocytes decreased expression of hexokinase, and reduced glucose uptake was seen in sAD [79]. In this study we observed similar changes in glycolytic pathways in astrocytes derived via an alternative reprogramming method, that maintains some markers of ageing, in both a sAD and fAD cases. Metabolic changes have also been associated with ageing; our data from the two control groups suggest differences with age in certain parameters, in particular hexokinase 1 mRNA expression, hexokinase 1 activity and complex I activity. However, further work with a wider ageing cohort would need to be undertaken to fully investigate this. In this study due to this potential age effect, we have always compared the sAD patient lines to the age matched control group and the PSEN1 familial group to age matched controls. Furthermore, we add to the literature with the finding that correcting the hexokinase 1 expression deficit in sAD astrocytes corrects the mis-localisation of the protein, and several of the glycolytic deficits seen, highlighting the therapeutic potential of upregulating hexokinase expression.

The literature surrounding bioenergetic changes in AD is not always consistent. In this work we have described deficits in both the glycolysis and OXPHOS pathways in AD astrocytes, but previous work has also shown increased glycolysis and mitochondrial function in sAD [79]. Cellular bioenergetic profile is likely affected by cell differentiation method, with iPSC derived cells not exposed to the epigenetic alterations that occur with age. In cell culture models the glucose concentration of cell media can differ between studies which may alter bioenergetic profiles. sAD is a heterogenous group of disease phenotypes with multiple factors effecting disease progression, unlike fAD which is largely the result of single gene mutations [79]. This may reflect why studies on cellular metabolism are not always consistent and highlights the importance of studying all aspects of disease pathophysiology in the sAD, so the AD cohort can be fully characterised and sub-divided.

Astrocytes shuttle lactate to neurons, with neurons preferring lactate over glucose at times of increased energy expenditure [80–82]. In this study, we have shown that astrocytes from both sporadic and familial AD patients have reduced extracellular lactate levels. Neurons rely mainly on OXPHOS to meet their energy requirements [38]; because of this they are at high risk of oxidative damage from free radicals produced via the ETC. Lactate preference, as an energy source, in neurons allows for the utilisation of neuronal glucose to produce antioxidant molecules such as glutathione via the pentose phosphate pathway (PPP) [82]. In non-neuronal cells, it has been identified that lactate can act as an activator of OXPHOS, and suppressor of glycolysis, which could have important implications for the astrocyte/neuron relationship [83]. If the supply of astrocyte lactate to neurons is impaired, this could lead to an increase in oxidative damage in AD neurons due to glucose being diverted away from the PPP. Oxidative damage to neurons has been reported when AD astrocytes are co-cultured with non-AD neurons [78], and in *postmortem* studies of the AD brain [84–86]. We have also shown in this study that sAD and fAD astrocytes have higher levels of mitochondrial ROS when compared with controls. The increase in astrocyte ROS seen in this study combined with the reduced extracellular lactate could potentially explain the higher levels of ROS seen in AD *postmortem* brain samples and co-culture studies.

The reduction in astrocyte total cellular ATP we report is likely to affect astrocyte/neuronal crosstalk. ATP is a signalling molecule within the brain, and astrocyte-released ATP modulates synaptic activity [87–89]. It is thought that astrocyte-derived ATP can alter the survivability of a developing synapse via its actions on the P2Y receptor [90]. As synaptic loss is an early feature in AD, support of astrocyte metabolism to increase total cellular ATP may help to prevent this pathological process.

We report changes in mitochondrial function and morphology. Previous work on human derived fAD astrocytes has suggested that the OCR is higher than that of matched controls [78]. Trends towards deficits in MSRC have also been identified but have not been shown to be significant [91]. Both of these studies focus on the same 3 PSEN1 astrocyte lines. The changes in MSRC are consistent with what we found in the present study although, we have shown the MSRC deficit to be significant. The basal OCR in our study was significantly lower in fAD astrocytes; this contrasts with the finding described by Oksanen and colleagues [78]. The difference seen here may be explained by the fact that we have studied point mutations in the PSEN1 gene that cause AD, whereas the Oksanen group have studied astrocytes created from an exon-9 deletion PSEN1 model of AD.

The reduction in mitochondrial cellular ATP production when complex I substrates are supplied to astrocytes, suggests a primary deficit in OXPHOS, but when we investigated the function of complex I, II, and IV directly, no deficit in activity was identified. Interestingly, in sporadic AD astrocytes the activity of complex I was higher than comparative controls, and this may in part help to explain the increased ROS level seen in sAD astrocytes. The increase in Vmax of complex I with a reduction in complex I linked ATP production, suggest that the enzyme itself has full functionality, however it is unable to operate at a sufficiently high level to maintain ATP levels. This may be due to substrate supply from other metabolic pathways which are also defective. The mis-localisation of hexokinase 1 we report in sAD may also contribute to the reduction in mitochondrial ATP production identified. It has been previously shown that increased mitochondrial ROS levels can stimulate both glucose uptake and GLUT1 expression in myoblasts [92]. The increased ROS levels we report in this study may be a compensatory mechanism by the astrocyte ETC to increase glucose uptake.

This is the first study to show that astrocytic glycolytic reserve, and extracellular lactate correlate with scores on neuropsychological tests shown to be affected early in AD and may have emerged as a result of the aged phenotype of the astrocyte model system used. However, caution in interpreting this correlation is needed, as this is a small cohort of cell lines in which disease and control astrocyte lines were plotted together to gain a meaningful correlation. The recent study by Samanta et al. directly highlights how cognitive function can be preserved by maintaining mitochondrial function in AD, supporting the findings of the present study [23]. Further work on larger cohorts of sporadic AD patients is needed before robust conclusions about these correlations can be made.

By increasing the expression of hexokinase 1 we were able to reduce the deficits in total cellular ATP and reduce the mitochondrial ROS levels in sAD astrocytes. The reduction in mitochondrial ROS seen in this study also suggests that an element of the astrocyte mitochondrial dysfunction is secondary to, or dependent on, AD glycolysis not providing enough substrates for the ETC and/or the direct action of hexokinase 1 at the mitochondrial membrane.

Increasing the expression of hexokinase 1 in fAD astrocytes did not improve any of the markers of glycolytic function or mitochondrial structure and function. This is potentially explained by our observation that sAD astrocytes have a mis-localisation of Hexokinase 1, with less hexokinase localised to the mitochondria, which is not seen in fAD astrocytes. The Presenilin proteins are known to effect mitochondria function and structure, and this highlights how, although there are similarities between the familial and sporadic forms of the disease, there are clearly upstream pathophysiological differences that lead to clinical presentation.

The present study is limited by sample size however it is one of the larger studies investigating metabolic abnormalities in astrocytes derived from sporadic AD patients; the number of PSEN1 mutant lines used is in line with most published studies. Larger cohorts of both sAD and fAD astrocytes should be investigated to validate our findings. To this end there are also age differences between the fAD and sAD cohorts, and although we always compare each disease group to age matched controls, some of the parameters measured here may change with age. Hence, a study to fully investigate this would be able to elucidate if any of these changes are a combination of ageing mechanisms and disease related mechanisms. Further work is needed to identify the mechanism behind metabolic dysfunction in fAD astrocytes, particularly if this relates to the action of PSEN1 on the mitochondrial membrane. Further investigations of hexokinase 1 as a potential therapeutic target in sAD along with further mechanistic work to elucidate the importance of the mitochondrial hexokinase 1 pool is critical in establishing this. Finally, our study focuses on astrocytes in monoculture and, it would be important in future studies to investigate the functional relationship between astrocytes and neurons using co-culture methods.

## Conclusions

In this study we show that astrocytes derived from patients with sAD or fAD have deficits in both mitochondrial function and glycolysis. We show that overexpressing hexokinase 1 can correct several of the glycolytic deficits and the mis-localisation of hexokinase 1 in sAD astrocytes but not in astrocytes from presenilin fAD cases. These deficits correlate with scores on neuropsychological tests that show decline early in AD. This report highlights a potential new therapeutic target in sAD and the benefits from studying both sporadic and familial forms of the condition simultaneously, to clarify pathophysiological divergence and similarity between these disease forms.

## Supplementary information

Supplementary tables and figures

## Data Availability

The authors confirm that the data supporting the findings of this study are available within the article and its supplementary materials. Additional data and information is available on request from the authors.
