# Supplementary material for: Increasing hexokinase 1 expression improves mitochondrial and glycolytic functional deficits seen in sporadic Alzheimer’s disease astrocytes
Source: Mol Psychiatry. 2024 Sep 13;30(4):1369–82. doi: 10.1038/s41380-024-02746-8 (PMC11919762; doi:10.1038/s41380-024-02746-8)
Supplement: Supplementary file 1 — Supplementary tables and figures [file 41380_2024_2746_MOESM1_ESM.pdf]

Increasing Hexokinase 1 expression improves mitochondrial and glycolytic functional deficits seen in sporadic Alzheimer's disease astrocytes.

Simon M Bell[1,2,3], Hollie Wareing[1], Francesco Capriglia[1], Rachel Hughes[1], Katy Barnes[1], Alexander Hamshaw[1], , Liam Adair[4,5], Allan Shaw[1], Alicja Olejnik[1], Suman De[1], Elizabeth New[4,5], Pamela J Shaw[1,2,3], Matteo De Marco[6], Annalena Venneri[6,7], Daniel J Blackburn[1,2], Laura Ferraiuolo[1,3], Heather Mortiboys[1,3*]

Institutions

1. **Sheffield Institute for Translational Neuroscience, School of Medicine and Population Health, University of Sheffield, 385a Glossop Rd, Sheffield S10 2HQ, UK**
2. **NIHR Sheffield Biomedical Research Centre, University of Sheffield and Sheffield Teaching Hospitals NHS Foundation Trust, Sheffield, UK**
3. **Neuroscience Institute, University of Sheffield, Firth Court, Sheffield, S10 2TN, UK**
4. **School of Chemistry, The University of Sydney, Sydney, NSW 2006, Australia**
5. **Australian Research Council Centre of Excellence for Innovations in Peptide and Protein Science, The University of Sydney, Sydney, NSW 2006, Australia**
6. **Department of Life Sciences, Brunel University London, Uxbridge, UK**
7. **Department of Medicine and Surgery, University of Parma, Parma, Italy**

Corresponding Authors:

Professor Heather Mortiboys

Professor of Cellular Neuroscience and Metabolism

Sheffield Institute for Translational Neuroscience (SITraN),

School of Medicine and Population Health,

University of Sheffield,

385a Glossop Road,

Sheffield

S10 2HQ, UK

H.Mortiboys@sheffield.ac.uk

Dr Simon M Bell

NIHR Clinical Lecturer in Neurology

Sheffield Institute for Translational Neuroscience (SITraN),

School of Medicine and Population Health,

University of Sheffield,

385a Glossop Road,

Sheffield

s.m.bell@sheffield.ac.uk

This supplementary file contains additional figures and tables that are referred to in the main body of the text of the article.

Supplementary Tables and Figures

Supplementary table 1| *Patient Demographic Information for sporadic AD lines and contemporary treatment status* #For control 7 some items of the MMSE could not be tested due to sensory impairment.

Patient Details

Sporadic AD (sAD) patients and matched controls were recruited as part of the MODEL-AD study (Yorkshire and Humber Research and Ethics Committee number: 16/YH/0155) for fibroblast biopsy with informed consent provided by all participants. All sAD and matched controls had previously participated in the European EU-funded Framework Programme 7 Virtual Physiological Human: Dementia Research Enabled by IT (VPH-DARE@IT) initiative (http://www.vph-dare.eu/). A diagnosis of Alzheimer's disease was made in sAD patients based on clinical guidelines (2).

Presenilin 1 AD patient fibroblasts (fAD) and matched controls were acquired from the NIGMS Human Genetic Cell Repository at the Coriell Institute for Medical Research under MTA agreement with HM: (ND41001, ND34733, AGO6848, GMO2189), and a cohort from a Sheffield based study (cohort lines 155 and 161). All fAD patients had a confirmed pathological mutation in the presenilin 1 gene (See Table 2). For all experiments, separate control groups for sAD and fAD were used.

All experiments carried out were approved by the Research and Ethics Committee or cell provider and methods were carried out in accordance with these guidelines.

**Patient Line**	**Age (Years)**	**Sex**	**MMSE**	**Length of Education (Years)**	**AD Treatment (at biopsy)**
**1**	60	Male	18	9	None
**2**	59	Female	23	11	Galantamine
**3**	63	Female	26	10	Donepezil
**4**	60	Male	18	11	Memantine
**5**	60	Male	18	11	None
**6**	79	Female	28	15	None
**Group Mean (Standard Dev)**	**63.5 (7.71)**		**21.83 (4.48)**	**11.16 (2.04)**	
**Control lines**					
**1**	52	Male	27	16	NA
**2**	61	Female	30	NA	NA
**3**	over 90	Female	24#	14	NA

**4**	52	Female	29	17	NA
**5**	56	Male	24	11	NA
**6**	73	Female	26	12	NA
**7**	75	Female	28	18	NA
**Group Mean (Standard Dev)**	**66.7 (17.3)**		**27.33 (2.16)**	**14.66 (2.80)**	

Supplementary table 2| ***Patient demographics for Familial AD presenilin 1 lines and associated controls***

Control Line	Age (Years)	Sex	Presenilin 1 Mutation
**Sheffield 161**	**31**	**Male**	**NA**
**Sheffield 155**	**40**	**Male**	**NA**
**Coriell (GM02189)**	**63**	**Male**	**NA**
**Group Mean (Standard Dev)**	**44.66 (16.5)**		
**Familial Line**	**Age (Years)**	**Sex**	**Presenilin 1 Mutation**
**Coriell (ND41001)**	47	Female	14q24.3<br>Intron 4, G deletion
**Coriell (ND34733)**	60	Male	P264L
**Coriell (AG06848)**	56	Female	missense mutation {Ala246Glu (A246E)
**Group Mean (Standard Dev)**	**48.33 (11.06)**		

Supplementary Table 3 | ***Antibodies used for Immunochemistry and western blotting.***

In this table details of antibodies used to characterise both iNPCs and astrocytes are displayed with supplier and dilution at which they were applied to the cells.

Immunohistochemistry method Astrocytes or fibroblasts were permeabilised and blocked in a solution of 0.2% Triton, PBS, and 5% horse serum for 1 hour at room temperature. Cells were then incubated with primary antibodies (Supplementary table 1) overnight with 5% horse serum and PBS. Following this, cells were washed with PBST once and PBS twice. Secondary antibodies were added at a concentration of 1:1000 for 1 hour, then Hoechst dye (Life

Technologies) added at a concentration of 1:5000 for 10minutes. Staining was visualised using an Opera Phenix high content imaging system (Perkin Elmer). All staining was imaged using the x40 water objective, with at least 10 fields of view per well per condition imaged. Quantification was carried out across at least 300 cells per technical repeat and across three biological repeats per control or patient line measured. Therefore, for each line at least 900 cells are included in the quantification data presented.

Western blot method Westerns were performed as previously described (1). 20µg of protein was loaded per sample. Membranes were probed for glucose transporter 1, 2 and 4, hexokinase 1. Membranes were also probed for GAPDH that was used as a loading control and with ATP5a as a control to assess the purity of the mitochondrially enriched fraction. Supplementary Table 3 illustrates the primary and secondary antibodies used for western blot analyses.

Bradford method for protein determination

5µl of bovine serum albumin (BSA) standard concentrations (100 µg/ml, 125µg/ml, 250µg/ml, 500µg/ml, 750µg/ml, 1000µg/ml, and 1200µg/ml) and dH_2O, as a negative control, were pipetted in triplicate into a clear 96 well plate, as well as 5µl of cell sample diluted 1:4 in dH_2O. 250µl coomassie blue was added to each well, and protein absorbance was measured using the PHERAstar plate reader (BMG Labtech). Linear regression analysis was applied using GraphPad Prism, and Microsoft Excel used to calculate protein concentrations in cell samples.

**Antibody**	**Species**	**Dilution**	**Supplier**
Vimentin	chicken	1:200	Abcam (AB5733)
Nestin	Mouse	1:200	Abcam (AB18102)
CD44	Rabbit	1:200	Abcam (AB157107)
S100β	Rabbit	1:200	Abcam (AB868)
PAX6	Rabbit	1:1000	Abcam (AB5790)
GFAP	Rabbit	1:200	Dako (Z0334)
EAAT2	Rabbit	1:200	Abcam (AB41621)
Lamin A/C	Mouse	1:800	Abcam (Ab40567)
**Secondary Antibodies**			
Alexia 488	Rabbit	1:1000	Invitrogen
Alexia 488	Mouse	1:1000	Invitrogen
Alexia 488	Chicken	1:1000	Invitrogen
Alexia 568	Rabbit	1:1000	Invitrogen
Alexia 568	Mouse	1:1000	Invitrogen
Alexia 674	Chicken	1:1000	Invitrogen
Alexia 674	Mouse	1:1000	Invitrogen
**Western Primary Antibodies**			
GLUT1	Rabbit	1:2000	Proteintech (66290-1-Ig)
GLUT2	Rabbit	1:250,500,1000	Abcam (ab54460)
GLUT4	Rabbit	1:250,500,1000	Abcam (ab33780)
Hexokinase 1	Rabbit	1:2000	Abcam (ab648)
GAPDH	Mouse	1:2000	Proteintech (60004-1-0g)
ATP5a	Mouse	1:1000	Abcam (ab14748)
**Secondary Western Antibodies**			
	Rabbit	1:5000	

	Mouse	1:10000	

Supplementary Table 4 | *qPCR technique and gene primer sequences*

qPCR Method For qPCR approximately 2,000,000 astrocytes were harvested. or 300,000 fibroblasts were harvested. RNA extraction was performed using the RNeasy Mini Kit (Qiagen) and complementary DNA (cDNA) synthesised using the QuantiTect reverse transcription kit (Qiagen), both as per the manufacturer's protocol. qPCR was performed using QuantiTect SYBR green PCR kit (Qiagen) with samples loaded at 12.5ng/µL per well, 25µL SYBR green, forward and reverse primers (250nM beta actin, RANBP17, LAMA3 and 400nM TERF2), made up with RNase free water to a total volume of 50µL. Supplementary Table 2 displays the forward and reverse primer sequences used.

Gene	Forward	Reverse	Supplier	Cells tested
Hexokinase 1 (HK1)	GTCCAAGAAGTCAGAGAT GCAGG	CTGCTGGTGAAAATCCGTA GTGG	Merck	Astrocytes
Beta Actin	TCCCCCAACTTGAGATGTA TGAAG	AACTGGTCTCAAGTCAGTG TACAGG	Merck	iAstrocytes Fibroblasts
RANBP17	CACTTCGATGCAGAGAGG CTA	CACTGGTTCCGACAGTCTTC	Merck	iAstrocytes Fibroblasts
TERF2	TTATTCGAGAAAAGAACTT GGCCC	TGAGGAGGTAGGGCTCGG	Merck	iAstrocytes Fibroblasts
LAMA3	TGTTTAAACTGCAGCCTCC CA	ACACATTTCAAGTTCCCGGC	Merck	iAstrocytes Fibroblasts

Supplementary Table 5 | *Astrocyte neuropsychological metabolic correlations* This table displays the correlations between different astrocyte metabolic parameters and neuropsychological test scores after controlling for participant age, brain reserve and years of education. For this analysis the sAD controls and sAD astrocytes were considered as one group. Numbers highlighted in bold are statistically significant correlations.

Neuropsychological Tests	Astrocyte Metabolic Marker	correlation	P-value
Immediate Episodic Recall			
	Mitochondrial Spare Respiratory Capacity	0.659	0.053
Delayed Episodic Recall			
	Mitochondrial Spare Respiratory Capacity	0.465	0.207
Immediate Episodic Recall			
	MMP	0.554	0.122
Delayed Episodic Recall			
	MMP	0.399	0.287
Immediate Episodic Recall			
	Glycolytic Reserve	0.766	**0.016**
Delayed Episodic Recall			
	Glycolytic Reserve	0.700	**0.036**
Immediate Episodic Recall			
	Extracellular Lactate Level	0.749	**0.020**
Delayed Episodic Recall			
	Extracellular Lactate Level	0.677	**0.045**

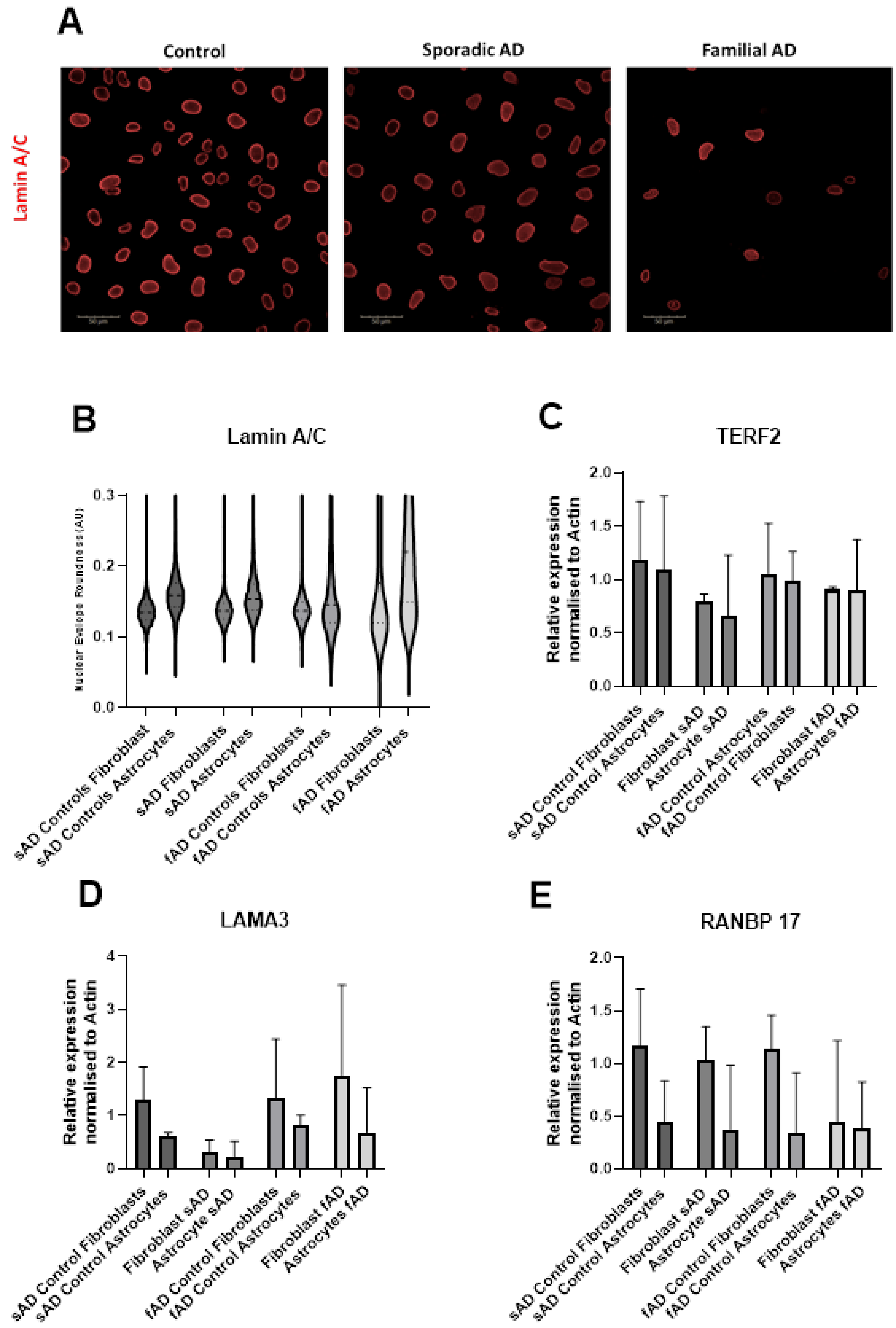
A
Control
Sporadic AD
Familial AD
Lamin A/C
B
Lamin A/C
Nuclear Envelope Roundness (AU)
sAD Controls Fibroblast
sAD Controls Astrocytes
sAD Fibroblasts
sAD Astrocytes
fAD Controls Fibroblasts
fAD Controls Astrocytes
fAD Fibroblasts
fAD Astrocytes
C
TERF2
Relative expression normalised to Actin
sAD Control Fibroblasts
sAD Control Astrocytes
Fibroblast sAD
Astrocyte sAD
fAD Control Astrocytes
fAD Control Fibroblasts
Fibroblast fAD
Astrocytes fAD
D
LAMA3
Relative expression normalised to Actin
sAD Control Fibroblasts
sAD Control Astrocytes
Fibroblast sAD
Astrocyte sAD
fAD Control Fibroblasts
fAD Control Astrocytes
Fibroblast fAD
Astrocytes fAD
E
RANBP 17
Relative expression normalised to Actin
sAD Control Fibroblasts
sAD Control Astrocytes
Fibroblast sAD
Astrocyte sAD
fAD Control Fibroblasts
fAD Control Astrocytes
Fibroblast fAD
Astrocytes fAD

Supplementary Figure 1 | Astrocyte Characterisation A Example images of Lamin A/C (red), staining of astrocytes. **B** Lamin A/C is a marker of aging of the nuclear envelope as described in our previous paper (3). This figure shows that in control astrocytes and fibroblasts there is no change in the shape of the nuclear envelope suggesting that the reprogramming of the cells has not returned them to a stem cell phase. As the protein is expressed at a low level in stem cells. **C-E** TERF2, LAMA3 and RANBP17 are all proteins that increase as a person ages (3). These figures show that the expression patterns of fibroblast and astrocyte levels of these proteins are similar for each line across cell types and similar to that which we have previously described in our previous paper(3). In each of the figures B-E the cell lines used are identical to those used for the hexokinase 1 transduction experiments.

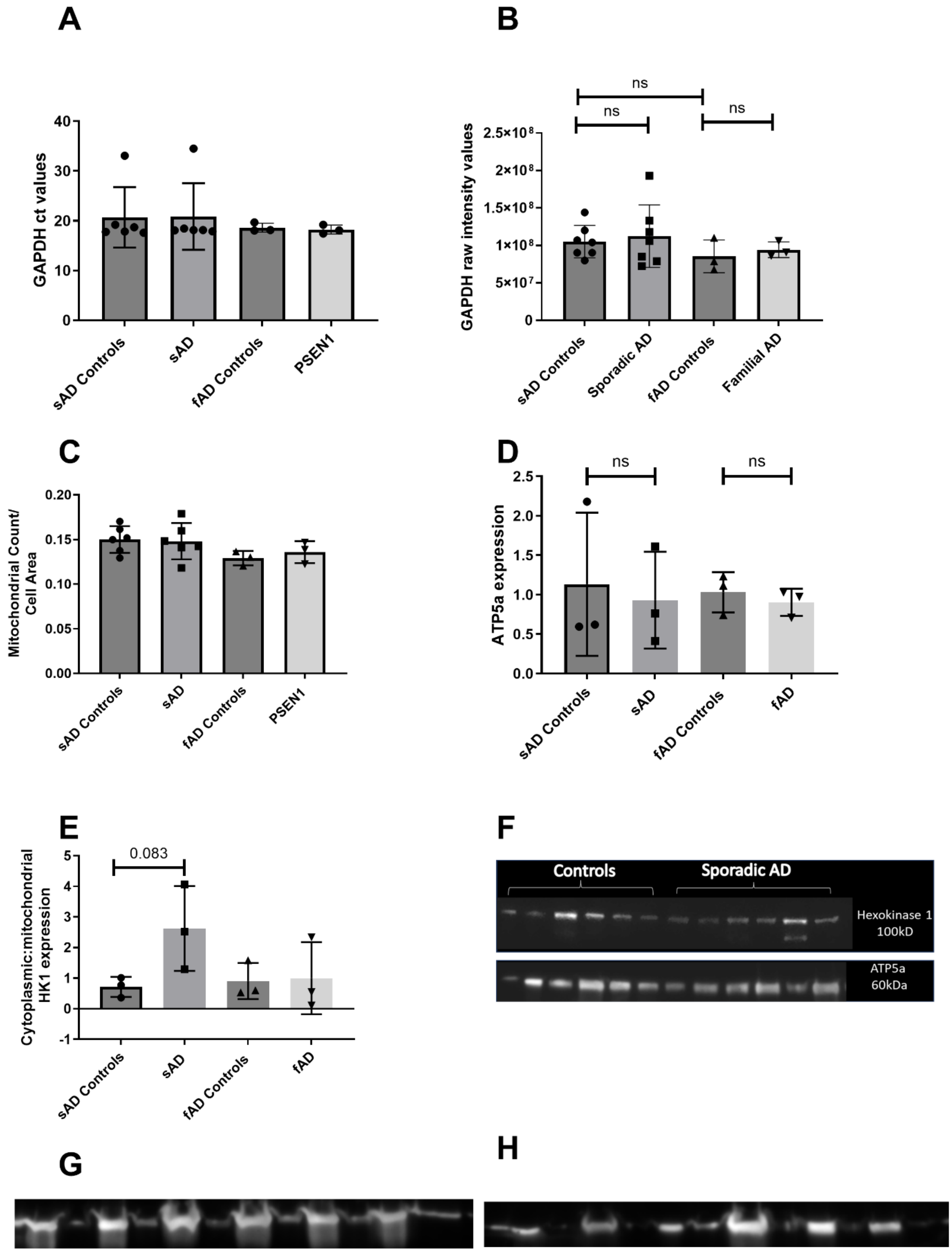
A
GAPDH ct values
0
10
20
30
40
sAD Controls
sAD
fAD Controls
PSEN1
B
GAPDH raw intensity values
ns
ns
ns
0
5×10⁷
1×10⁸
1.5×10⁸
2×10⁸
2.5×10⁸
sAD Controls
Sporadic AD
fAD Controls
Familial AD
C
Mitochondrial Count/
Cell Area
0.00
0.05
0.10
0.15
0.20
sAD Controls
sAD
fAD Controls
PSEN1
D
ATP5a expression
ns
ns
0.0
0.5
1.0
1.5
2.0
2.5
sAD Controls
sAD
fAD Controls
fAD
E
Cytoplasmic:mitochondrial
HK1 expression
0.083
-1
0
1
2
3
4
5
sAD Controls
sAD
fAD Controls
fAD
F
Controls
Sporadic AD
Hexokinase 1
100kD
ATP5a
60kDa
G
H

Supplementary Figure 2 | Relative expression of GAPDH, ATP5a and positioning of Hexokinase within the astrocyte **A** shows that a mRNA level the expression of GAPDH is not significantly different between control and AD astrocytes **B** Highlights that protein expression of GAPDH is not difference between AD and control astrocytes **C** Shows that when controlling for cell area mitochondrial number does not differ between astrocyte types **D** Highlights that the expression of the mitochondrial protein ATP5a also does not differ between astrocyte lines. **E** highlights that the in sAD astrocytes there is a greater proportion of HK1 within the cytosolic fraction of the cell when compared to controls. No difference is seen in fAD astrocyte HK1 cellular location when compared to controls **F** represents a representative blot for the mitochondrially enriched fraction experiments. The blot represents three control lines and three sAD. **G and H** are representative western blots showing the purity of the mitochondrial enriched fractions using ATP5a as a mitochondrial marker. **G** samples loaded fAD control1 mitochondria, fAD control1 cytoplasm, fAD control2 mitochondria, fAD control2 cytoplasm, fAD control3 mitochondria, fAD control3 cytoplasm, fAD PSEN1 A mitochondria, fAD PSEN1 A cytoplasm, fAD PSEN1 B mitochondria, fAD PSEN1 B cytoplasm, fAD PSEN1 C mitochondria and fAD PSEN1 C cytoplasm. **H** samples loaded sAD control 1 mitochondria, sAD control 1 cytoplasm, sAD control 2 mitochondria, sAD control 2 cytoplasm, sAD control 3 mitochondria, sAD control 3 cytoplasm, sAD patient 1 mitochondria, sAD patient 1 cytoplasm, sAD patient 2 mitochondria, sAD patient 2 cytoplasm, sAD patient 3 mitochondria, sAD patient 3 cytoplasm.

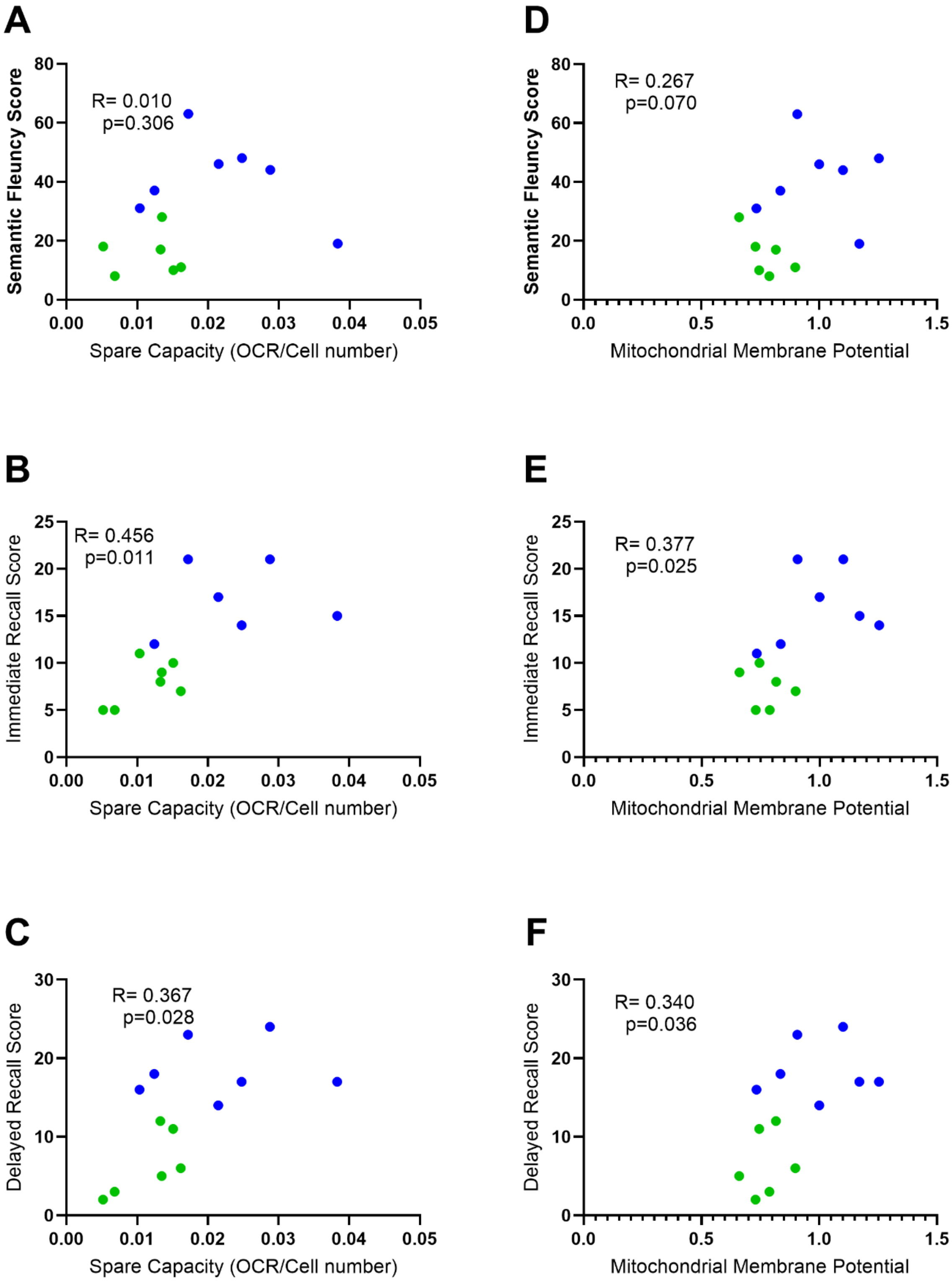

A
R= 0.010
p=0.306
Semantic Fleuncy Score
Spare Capacity (OCR/Cell number)
B
R= 0.456
p=0.011
Immediate Recall Score
Spare Capacity (OCR/Cell number)
C
R= 0.367
p=0.028
Delayed Recall Score
Spare Capacity (OCR/Cell number)
D
R= 0.267
p=0.070
Semantic Fleuncy Score
Mitochondrial Membrane Potential
E
R= 0.377
p=0.025
Immediate Recall Score
Mitochondrial Membrane Potential
F
R= 0.340
p=0.036
Delayed Recall Score
Mitochondrial Membrane Potential

Supplementary Figure 3 | *MSRC and MMP correlations with Neuropsychological tests*
This figure highlights the mitochondrial spare respiratory capacity (MSRC) correlations with semantic fluency **A** immediate episodic recall **B** and delayed episodic recall **C**. Mitochondrial membrane potential correlations (MMP) with semantic memory **D** immediate episodic recall **E** and delayed episodic recall **F** are also displayed. In each correlation 7 sAD controls and 6 sAD cell lines are plotted. The correlations are performed using a 2-tailed p-value and a Pearson correlation coefficient. Points in green represent sAD lines and those in blue represent sAD Controls.

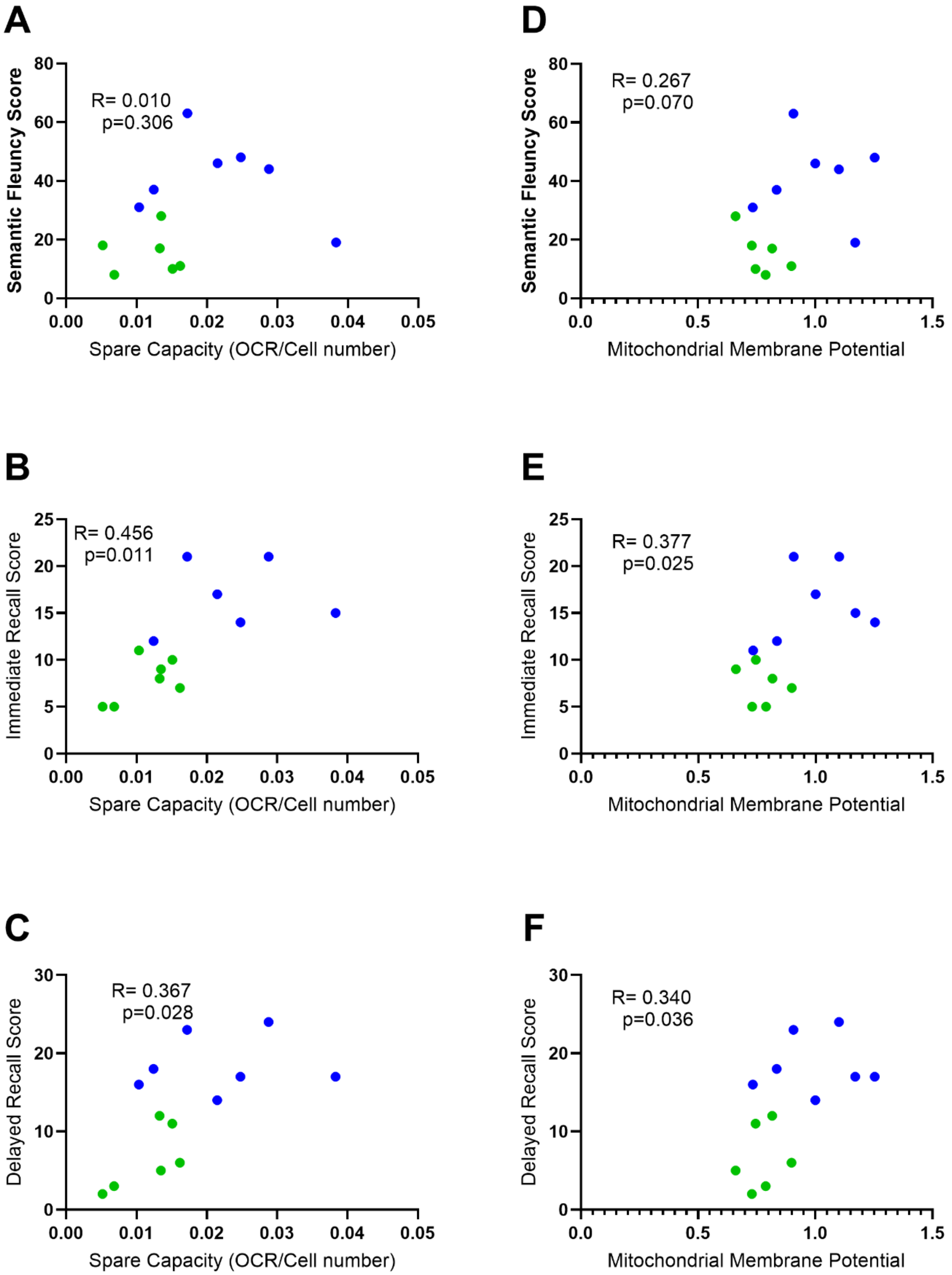
A
R= 0.010
p=0.306
Semantic Fleuncy Score
Spare Capacity (OCR/Cell number)
D
R= 0.267
p=0.070
Semantic Fleuncy Score
Mitochondrial Membrane Potential
B
R= 0.456
p=0.011
Immediate Recall Score
Spare Capacity (OCR/Cell number)
E
R= 0.377
p=0.025
Immediate Recall Score
Mitochondrial Membrane Potential
C
R= 0.367
p=0.028
Delayed Recall Score
Spare Capacity (OCR/Cell number)
F
R= 0.340
p=0.036
Delayed Recall Score
Mitochondrial Membrane Potential

Supplementary Figure 4 | ***Glycolytic reserve and extracellular lactate correlations with Neuropsychological tests*** This figure highlights the glycolytic reserve correlations with semantic fluency **A** immediate episodic recall **B** and delayed episodic recall **C.** Astrocyte extracellular lactate correlations with semantic memory **D** immediate episodic recall **E** and delayed episodic recall **F** are also displayed. In each correlation, 7 sAD controls and 6 sAD cell lines are plotted. The correlations are performed using a 2-tailed p-value and a Pearson correlation coefficient. Points in green represent sAD lines and those in blue represent sAD Controls.

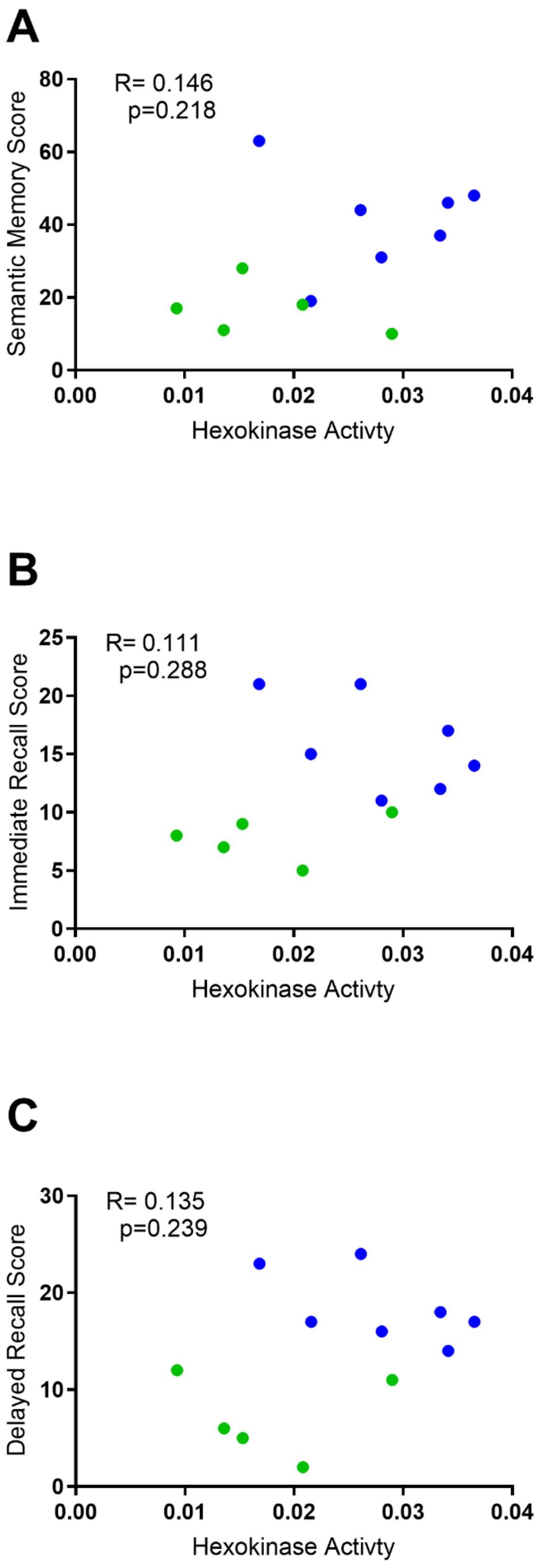
A
R= 0.146
p=0.218
Semantic Memory Score
80
60
40
20
0
0.00
0.01
0.02
0.03
0.04
Hexokinase Activty
B
R= 0.111
p=0.288
Immediate Recall Score
25
20
15
10
5
0
0.00
0.01
0.02
0.03
0.04
Hexokinase Activty
C
R= 0.135
p=0.239
Delayed Recall Score
30
20
10
0
0.00
0.01
0.02
0.03
0.04
Hexokinase Activty

Supplementary Figure 5 | *__Hexokinase 1 activity correlations with neuropsychological tests.__* This figure highlights the hexokinase 1 activity correlations with semantic fluency **A** immediate episodic recall **B** and delayed episodic recall **C.** In each correlation 7 sAD controls and 5 sAD cell lines are plotted. The correlations are performed using a 2-tailed p-value and a Pearson correlation coefficient. 5 sAD lines were included due to poor growth of the 6th line not allowing experimentation to be performed. Points in green represent sAD lines and those in blue represent sAD Controls.

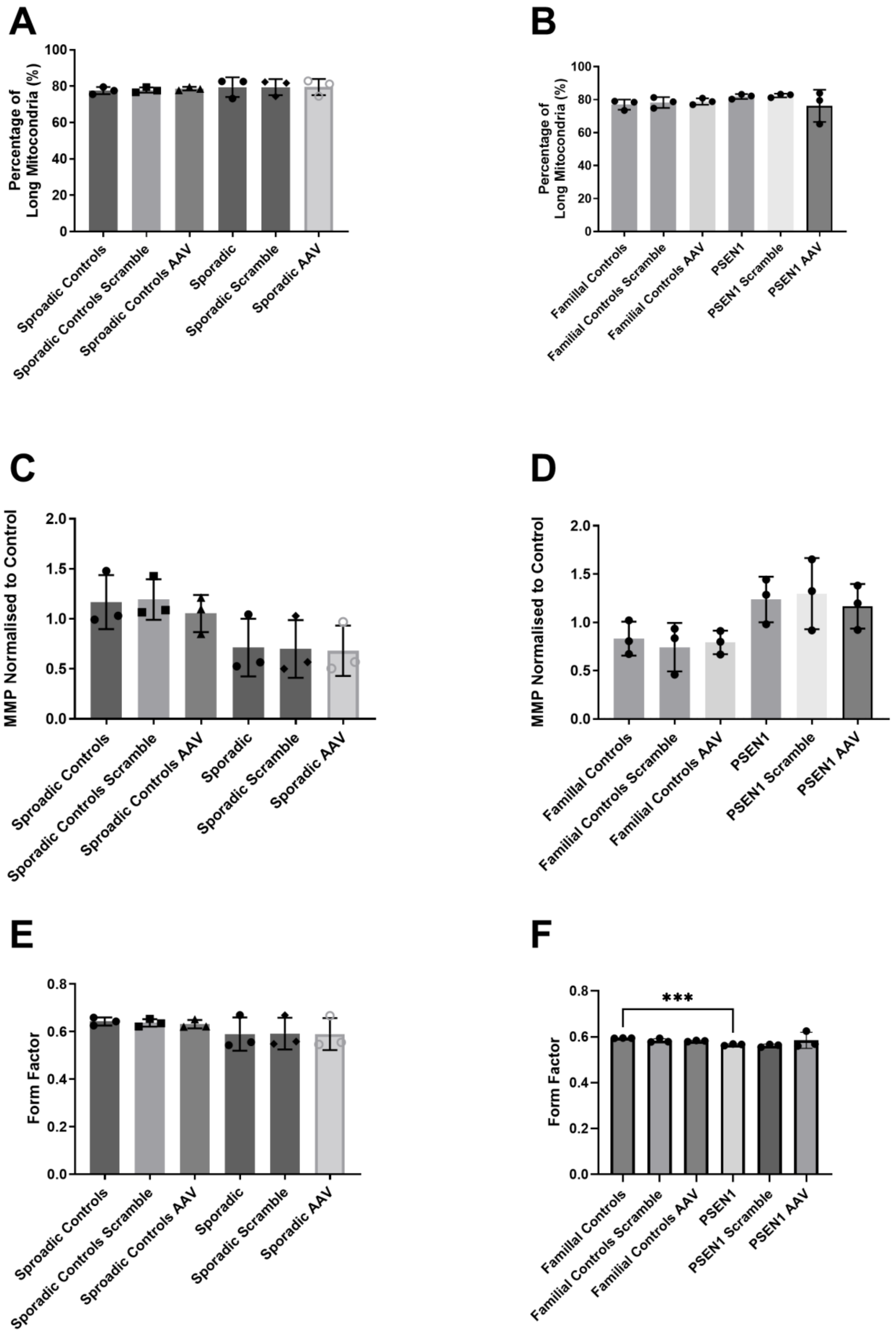
A
Percentage of Long Mitochondria (%)
Sproadic Controls
Sporadic Controls Scramble
Sproadic Controls AAV
Sporadic
Sporadic Scramble
Sporadic AAV
B
Percentage of Long Mitochondria (%)
Familial Controls
Familial Controls Scramble
Familial Controls AAV
PSEN1
PSEN1 Scramble
PSEN1 AAV
C
MMP Normalised to Control
Sproadic Controls
Sporadic Controls Scramble
Sproadic Controls AAV
Sporadic
Sporadic Scramble
Sporadic AAV
D
MMP Normalised to Control
Familial Controls
Familial Controls Scramble
Familial Controls AAV
PSEN1
PSEN1 Scramble
PSEN1 AAV
E
Form Factor
Sproadic Controls
Sporadic Controls Scramble
Sproadic Controls AAV
Sporadic
Sporadic Scramble
Sporadic AAV
F
Form Factor
***
Familial Controls
Familial Controls Scramble
Familial Controls AAV
PSEN1
PSEN1 Scramble
PSEN1 AAV

Supplementary Figure 6 | Mitochondrial morphological parameters after transduction with an AdV containing the hexokinase 1 gene. **A** sAD MMP values, **B** sAD long mitochondrial proportion. **C** sAD Form factor. **D** fAD MMP values, **E** fAD long mitochondria proportion, **F** fAD Form factor. In all experiments AD astrocytes are compared with matched controls using t-tests. Each experiment included 3 sAD controls, 3 sAD lines, 3 fAD control lines and 3 fAD lines. Data were analysed after at least 3 technical repeats and after 3 biological repeats were performed in each experiment.

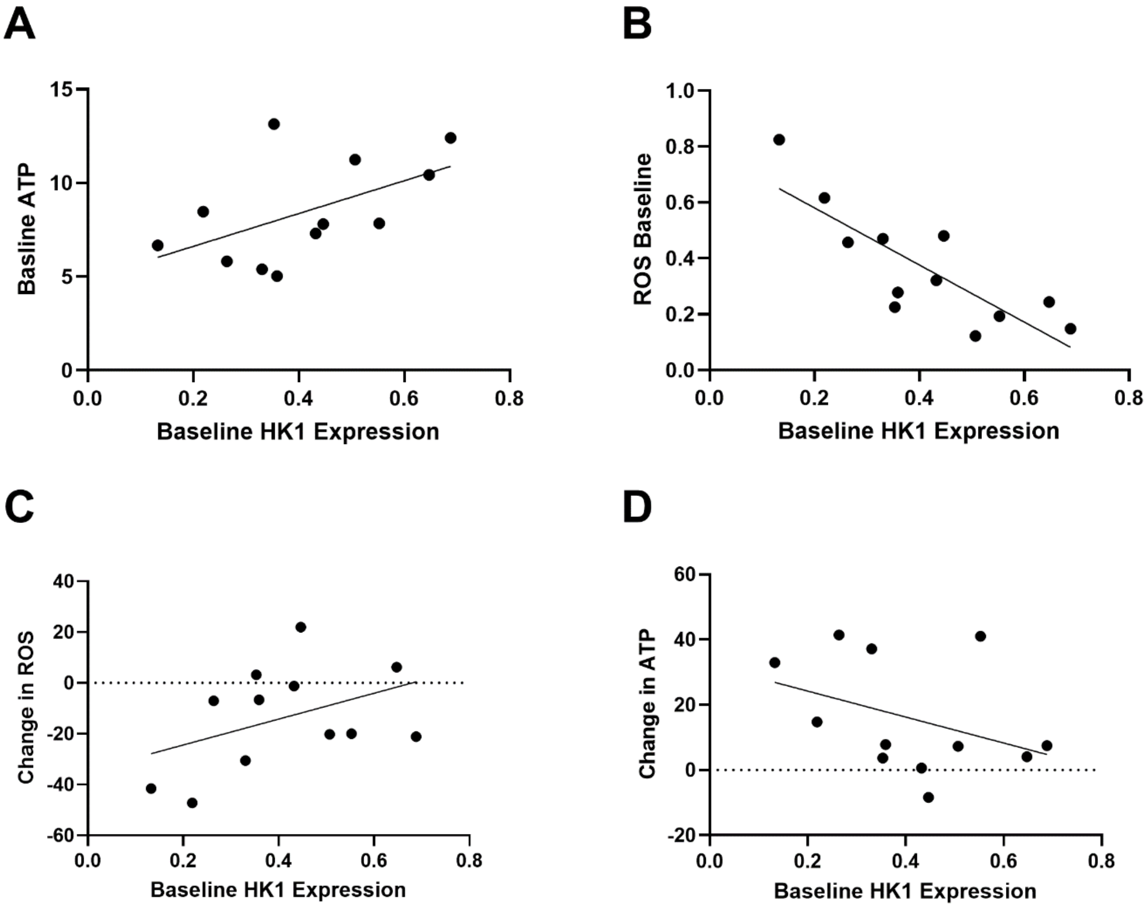
A
B
C
D
Basline ATP
ROS Baseline
Change in ROS
Change in ATP
Baseline HK1 Expression
0.0
0.2
0.4
0.6
0.8
1.0
15
10
5
0
40
20
-20
-40
-60
60

Supplementary Figure 7 | <u>Correlations of ATP and mitochondrial ROS with hexokinase 1 expression.</u> This figure highlights the hexokinase 1 expression level correlations with cellular ATP and mitochondrial ROS. **A** baseline ATP levels **B** baseline mitochondrial ROS **C** change in ATP levels after HK1 overexpression and **D** change in mitochondrial ROS after HK1 overexpression**.** In each correlation 7 sAD controls and 5 sAD cell lines are plotted. The correlations are performed using a 2-tailed p-value and a Pearson correlation coefficient. 5 sAD lines were included due to poor growth of the 6th line not allowing experimentation to be performed.

References

1. Bell SM, Barnes K, Clemmens H, Al-Rafiah AR, Al-Ofi EA, Leech V, et al. Ursodeoxycholic Acid Improves Mitochondrial Function and Redistributes Drp1 in Fibroblasts from Patients with Either Sporadic or Familial Alzheimer's Disease. Journal of molecular biology. 2018;430(21):3942-53.
2. McKhann GM, Knopman DS, Chertkow H, Hyman BT, Jack CR, Jr., Kawas CH, et al. The diagnosis of dementia due to Alzheimer's disease: recommendations from the National Institute on Aging-Alzheimer's Association workgroups on diagnostic guidelines for Alzheimer's disease. Alzheimer's & dementia : the journal of the Alzheimer's Association. 2011;7(3):263-9.
3. Gatto N, Dos Santos Souza C, Shaw AC, Bell SM, Myszczynska MA, Powers S, et al. Directly converted astrocytes retain the ageing features of the donor fibroblasts and elucidate the astrocytic contribution to human CNS health and disease. Aging Cell. 2021;20(1):e13281.
